# Sortilin‐Mediated Rapid, Precise and Sustained Degradation of Membrane Proteins via mRNA‐Encoded Lysosome‐Targeting Chimera

**DOI:** 10.1002/advs.202501222

**Published:** 2025-04-30

**Authors:** Xin Chang, Xinyu Qiu, Xiaoning Tong, Shaoju Gan, Weicheng Yi, Sitao Xie, Xiangsheng Liu, Chao Zuo, Weihong Tan

**Affiliations:** ^1^ Molecular Science and Biomedicine Laboratory (MBL) State Key Laboratory of Chemo/Biosensing and Chemometrics College of Chemistry and Chemical Engineering College of Biology Hunan University Changsha Hunan 410082 China; ^2^ Zhejiang Cancer Hospital Hangzhou Institute of Medicine (HIM) Chinese Academy of Sciences Hangzhou Zhejiang 310022 China; ^3^ Department of General Surgery Fujian Medical University Union Hospital Fuzhou Fujian 350001 China

**Keywords:** cancer therapy, molecular engineering, mRNA, precision medicine, protein degradation

## Abstract

Recent advances in lysosome‐targeting degradation technologies have introduced strategies to regulate therapeutic membrane proteins (MPs), potentially transforming treatment paradigms. However, challenges persist, including limited degradation precision due to the broad distribution of lysosome‐targeting receptors (LTRs), as well as the high cost and complexity of recombinant protein production or chemical synthesis. Herein, it identifies sortilin as a promising LTR, highly expressed in malignancies but minimally present in healthy tissues outside the nervous system. Using AlphaFold‐Multimer, it screened for a specific non‐endogenous protein binder to sortilin and developed a modular, mRNA‐encoded lysosomal targeting chimera (MedTAC) strategy, enabling rapid design and precise degradation of oncogenic MPs. In a breast cancer‐bearing mouse model, a single low dose of MedTAC_PTK7_ (0.5 mg kg^−1^) reduced protein tyrosine kinase‐7 (PTK7) levels by up to 80% within 24 h, with sustained degradation of 44% at 72 h, demonstrating excellent pharmacokinetics. MedTAC_PTK7_ significantly extended survival to over 50 days without systemic toxicity, compared to 20–30 days in controls. This MedTAC strategy establishes sortilin as a selective and efficient shuttle for targeted protein degradation, offering a scalable, rapidly producible platform for biochemical research and precise therapeutic applications.

## Graphical abstract

The study identified sortilin as a promising LTR, enabling targeted degradation of oncogenic proteins through an mRNA‐encoded MedTAC strategy. In a mouse model, MedTAC_PTK7_ reduced PTK7 by up to 80%, extended survival, and showed excellent pharmacokinetics without toxicity, providing a scalable platform for targeted therapies.

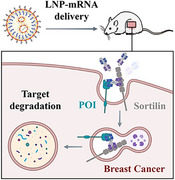

## 1 Introduction

MPs constitute ≈23% of the human proteome and account for over 60% of therapeutic targets, underscoring their crucial roles in diseases such as autoimmune disorders, neurodegenerative conditions and cancers.^[^ ^1^ ^]^ For instance, activation of the epidermal growth factor receptor (EGFR) pathway promotes cell proliferation and metastasis in many cancers,^[^ ^2^ ^]^ while overexpression of programmed death‐ligand 1 (PD‐L1) enables cancer cells to evade immune surveillance.^[^ ^3^ ^]^ Advances in modulating MPs functions and interactions have not only deepened our understanding of their biological mechanisms but also opened new avenues for innovative therapies.

Among recent advancements, lysosome‐targeting degradation has emerged as a promising strategy, providing a more controllable and rapid‐response approach compared to traditional gene‐editing techniques.^[^ ^4^ ^]^ Lysosome‐targeting chimeras (LYTACs) function as bifunctional molecules, binding both MPs and lysosome‐targeting receptors (LTRs) to direct the targeted proteins toward lysosomal degradation. For example, LYTACs utilizing the mannose‐6‐phosphate/insulin‐like growth factor II receptor (M6P/IGFIIR)^[^ ^5^ ^]^ and the asialoglycoprotein receptor (ASGPR)^[^ ^5^ ^]^ have successfully facilitated the degradation of both membrane and extracellular proteins. Other lysosomal shuttle receptors, including cytokine receptors,^[^ ^6^ ^]^ transferrin receptors,^[^ ^7^ ^]^ scavenger receptors,^[^ ^8^ ^]^ and integrins,^[^ ^9^ ^]^ have also been employed in receptor‐ligand‐mediated lysosomal degradation systems. However, achieving precise degradation of MPs, particularly in extrahepatic tissues, remains challenging due to the unavoidable expression of proteins of interest (POIs) on healthy cells and the broad distribution of LTRs (Table S1, Supporting Information). This highlights the urgent need to develop tissue‐ or cell‐specific LTRs to enable targeted degradation strategies that selectively eliminate POIs while minimizing systemic toxicity.^[^ ^5a^ ^]^

In addition, LYTACs and other lysosome‐targeting degradation technologies face several challenges, including suboptimal pharmacokinetics, design complexity, and labor‐intensive production processes, which hinder their broader clinical application.^[^ ^5^, ^10^ ^]^ For instance, the production of M6Pn‐LYTACs and GalNAc‐LYTACs involves intricate glycan cluster synthesis and extensive antibody engineering, leading to higher costs and scalability challenges.^[^ ^11^ ^]^ While aptamer‐based LYTACs offer simpler and more precise synthesis, they still encounter issues with reduced chemical stability and shorter half‐lives.^[^ ^9^, ^12^ ^]^ Recently, recombination‐based and modular lysosome‐targeting technologies have emerged as more scalable approaches for MPs degradation.^[^ ^6^, ^7^, ^10^, ^13^ ^]^ Nevertheless, recombinant protein therapies continue to struggle with high production costs and the risks of protein misfolding or aggregation.^[^ ^14^ ^]^ In this context, strategies that enable rapid development and optimize pharmacokinetics without compromising potency are highly desired.

**Scheme 1:**
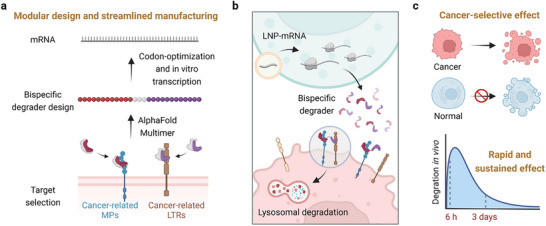
Schematic illustration of the MedTAC‐mediated rapid, precise, and sustained degradation of membrane proteins (MPs). a) Modular design and preparation of MedTAC. Sortilin was identified as a cancer‐related LTR through bioinformatics analysis. Using the AlphaFold‐Multimer algorithm, protein binders specifically targeting Sortilin or MPs were developed, subsequently designed as bispecific degraders and encoded into sequence‐optimized, chemically modified mRNA. b) Lipid nanoparticles (LNP) encapsulated MedTAC were delivered into cells, enabling sustained expression of bispecific degraders to mediate degradation of specific membrane proteins on tumor cell surfaces in a Sortilin‐dependent manner. c) In a breast cancer‐bearing mouse model, MedTAC significantly inhibited tumor growth without systemic toxicity, achieving rapid and sustained degradation of MPs in vivo.

mRNA‐based therapies provide streamlined production and improved pharmacokinetics, demonstrating superior efficacy in certain clinical contexts compared to recombinant proteins.^[^ ^15^ ^]^ Inspired by these benefits, we introduce a novel mRNA‐encoded lysosomal targeting chimera (MedTAC) strategy to degrade cancer‐related MPs (**Scheme** **1**). In this work, sortilin is selected as a cancer‐related LTR, due to its efficient lysosomal transport capabilities, high expression in cancers, and limited distribution in normal tissues outside the nervous system.^[^ ^16^ ^]^ Using the AlphaFold‐Multimer algorithm,^[^ ^17^ ^]^ we identified specific protein binders to direct MPs to the lysosome in a sortilin‐dependent manner. Our results show that sortilin‐mediated lysosomal degradation is both effective and sustained in vivo (Scheme 1). Low‐dose administration of the PTK7‐targeting MedTAC (MedTAC_PTK7_) resulted in a sustained reduction of PTK7 in breast cancer‐bearing mice, maintaining a 44% degradation rate three days post‐treatment, without significant systemic toxicity. Overall, this study underscores the therapeutic potential of MedTAC, emphasizing its specificity, ease of production and favorable pharmacokinetics, paving the way for precise and effective MPs degradation.

## 2 Results

### 2.1 Characterization of Sortilin and Its Specific Protein Binders

**Figure 1:**
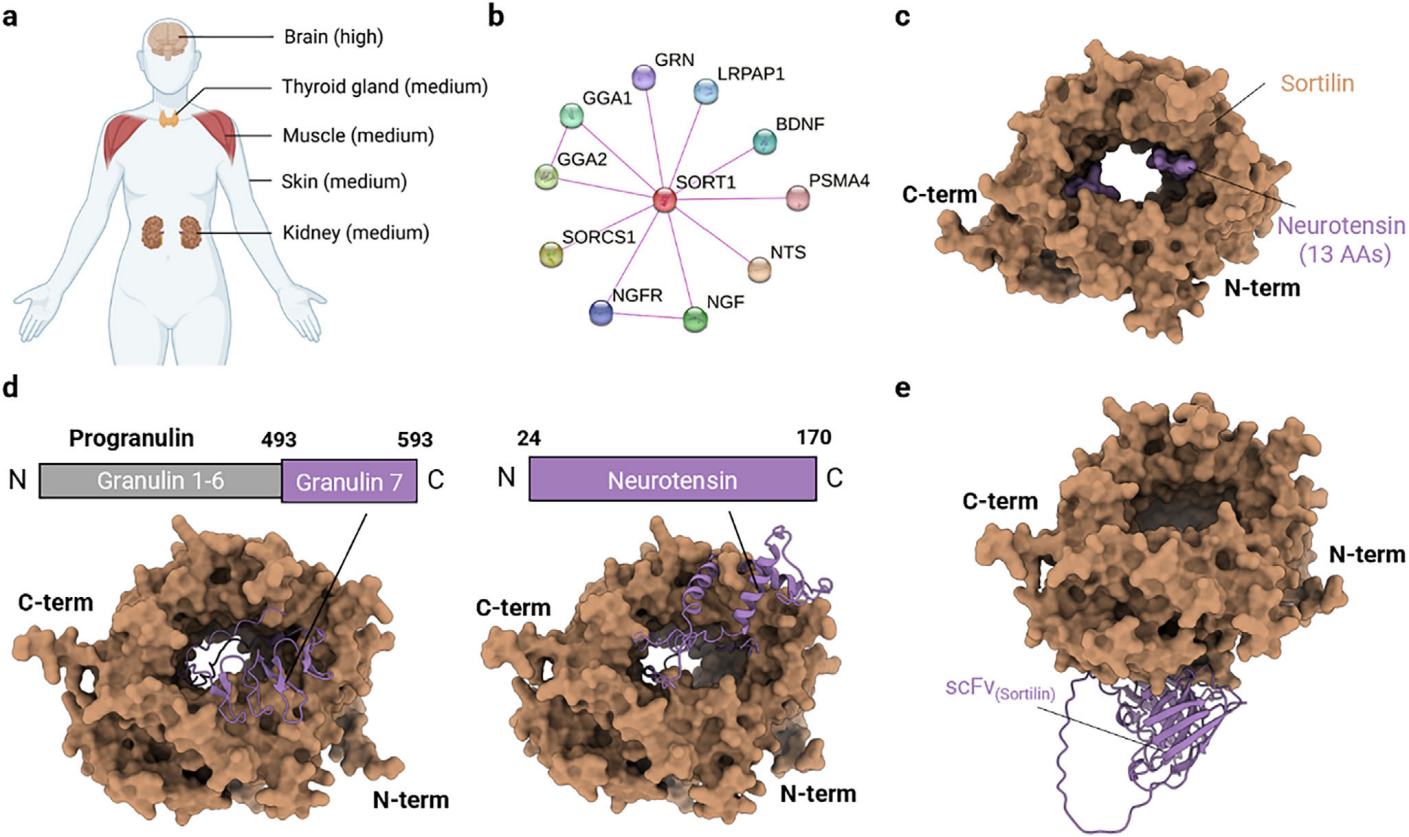
Overview of sortilin characterization and targeted binder screening. a) Tissue distribution map of sortilin in the human body, highlighting high expression levels in the brain and moderate expression in the thyroid gland, muscle, skin, and kidney. b) Analysis of the PPI network associated with sortilin. c) AlphaFold‐Multimer predictions for the PGRN_493‐593_/sortilin and neurotensin_24‐170_/sortilin complexes, with sortilin in light brown and progranulin or neurotensin in purple brown. d) Structure overview of the tridecapeptide/sortilin complex, with sortilin shown in light brown and the tridecapeptide in purple brown (PDB: 4PO7). e) AlphaFold‐Multimer predictions of the designed scFv_(sortilin)_ binding to the external surface of sortilin's tunnel conformation, with sortilin in light brown and scFv_(sortilin)_ in purple brown.

Sortilin acts as a critical clearance receptor on the cell surface, regulating protein trafficking through endocytosis. This mechanism enables the rapid internalization and lysosomal transport of ligands like neurotensin (NTS) and progranulin (PGRN).^[^ ^18^ ^]^ According to the Human Protein Atlas (HPA),^[^ ^19^ ^]^ sortilin is primarily expressed in the brain, with moderate expression in the thyroid gland, muscle, skin and kidney (**Figure** **1**a). This expression profile makes sortilin particularly suitable for liver‐enriched drugs, such as lipid nanoparticles (LNP) encapsulated mRNA therapeutics, given its relatively low expression in the liver. Furthermore, sortilin is significantly overexpressed in several cancers, including breast, colorectal, ovarian, pancreatic, melanoma and pituitary adenoma, highlighting its potential as a therapeutic target.^[^ ^20^ ^]^ Compared to other LTRs, sortilin's efficient lysosomal transport capabilities and specific expression profile position it as a promising candidate for targeted therapies (Table S1, Supporting Information). During the review of this manuscript, Huang et al. conducted a preliminary test of sortilin as an LTR for EGFR degradation in vitro.^[^ ^13^ ^]^ Here, we further investigate sortilin's potential as an LTR through comprehensive in vitro and in vivo experiments.

To identify specific protein binders for sortilin, we analyzed its protein‐protein interaction (PPI) network using the STRING database (Figure 1b).^[^ ^21^ ^]^ Among the experimentally validated ligands, we identified NTS, PGRN, beta‐nerve growth factor (NGF) and brain‐derived neurotrophic factor (BDNF) as extracellular proteins. We employed the AlphaFold‐Multimer algorithm to predict and analyze the interactions between these ligands and sortilin (Figure 1c; Figure S1a,b, Supporting Information). The conformations and interaction patterns of the NTS_24‐170_/sortilin complex closely resembled those of the tridecapeptide/sortilin complex,^[^ ^22^ ^]^ with a root‐mean‐square deviation (RMSD) of 0.574 Å (Figure 1d). Furthermore, NTS, PGRN, NGF, and BDNF all occupy the tunnel of the *β*‐propeller domain of sortilin, suggesting a possible shared molecular mechanism through which these endogenous ligands might regulate sortilin signaling (Figure 1c; Figure S1a,b, Supporting Information). In subsequent experiments, we did not test all four endogenous ligands. We selected PGRN for further study because it exhibited the highest predicted Local Distance Difference Test (pLDDT), predicted aligned error (pAE) and Interface Predicted Template Modeling (ipTM) score (Figure S1c and Table S2, Supporting Information).

To design a protein binder that do not interfere with sortilin's function, we selected Latozinemab,^[^ ^23^ ^]^ a human monoclonal antibody with high specificity for sortilin. Full‐length IgG typically requires the assembly of two different proteins, each encoded by separate mRNAs.^[^ ^15c^ ^]^ To streamline production, we developed a single‐chain variable fragment called scFv_(sortilin)_ from Latozinemab, which requires only a single mRNA for encoding. Structural modeling of the scFv_(sortilin)_/sortilin complex indicated that scFv_(sortilin)_ binds to the external surface of the tunnel conformation, distinct from the binding sites of endogenous protein ligands (Figure 1e). These findings suggest that scFv_(sortilin)_ may serve as a specific sortilin‐targeting protein binder without disrupting its physiological activity.

### 2.2 Design of MedTAC for PTK7 Degradation

**Figure 2:**
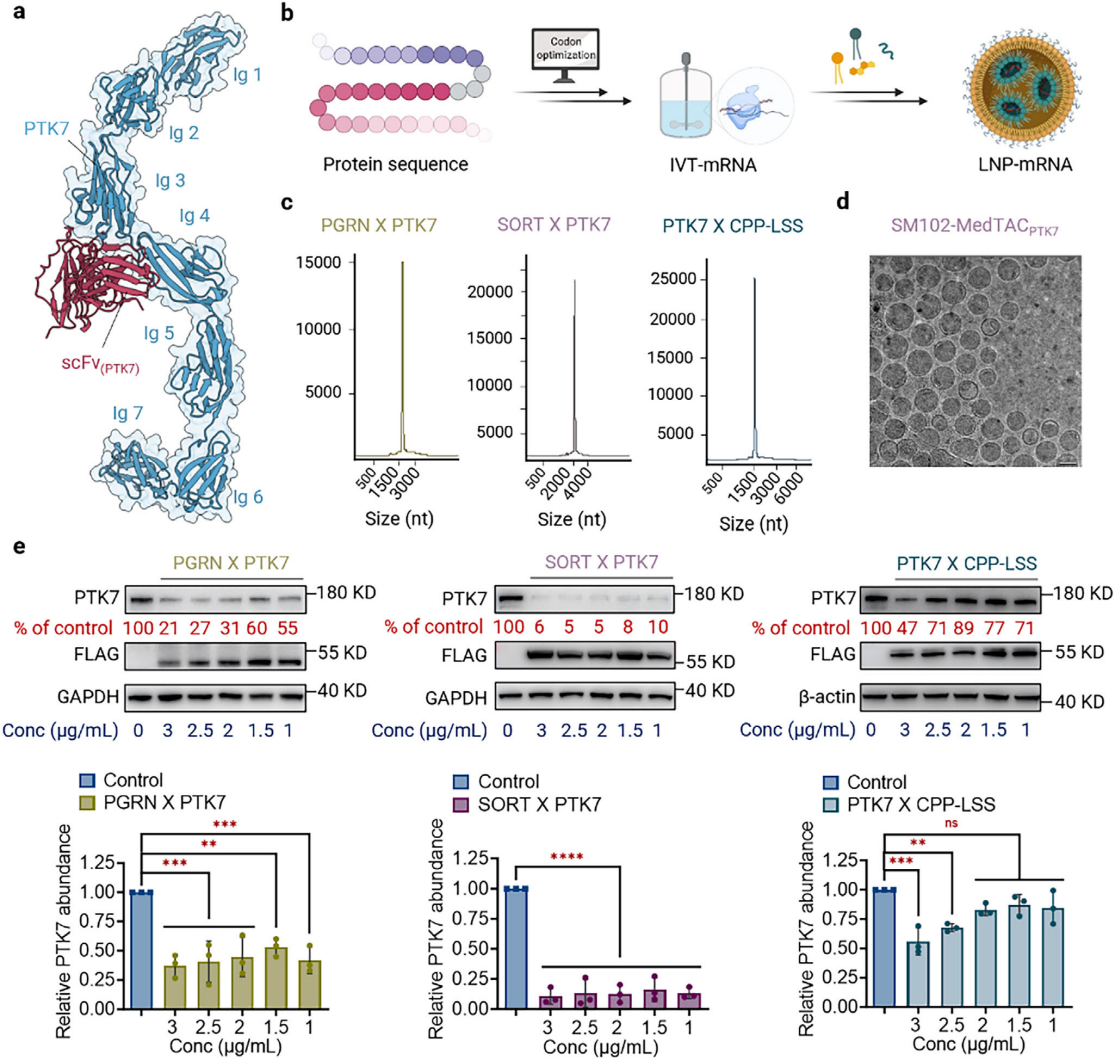
Development and evaluation of MedTAC_PTK7_ for targeted PTK7 degradation. a) AlphaFold‐Multimer predictions showing the interaction between scFv(_PTK7_), derived from Cofe‐P, and the extracellular domain of PTK7, with PTK7 depicted in light blue and scFv(_PTK7_) in deep red. b) Flowchart outlining the MedTAC preparation process, including protein sequence design, in vitro mRNA transcription and LNP encapsulation. c) Verification of mRNA integrity through capillary gel electrophoresis. d) Transmission electron microscopy images of LNP‐encapsulated mRNAs. e) Western blot analysis of PTK7 levels in MDA‐MB‐468 cells treated with gradient concentrations of PGRN × PTK7, SORT × PTK7 and PTK7 X CPP‐LSS for 48 h, presented as mean ± SD (n = 3). All the p values were determined by ordinary one‐way ANOVA. n.s., not significant; ****^p^ < 0.001, ***^p^ < 0.01, **^p^ < 0.05.

With the identified sortilin binders in hand, we explored the potential of MedTAC technology for selectively targeting and degrading MPs. We selected PTK7 as the target due to its high expression in both primary and metastatic tumors, which is strongly associated with tumor progression, metastasis and poor prognosis.^[^ ^24^ ^]^ To target PTK7, we designed a scFv_(PTK7)_ derived from Cofetuzumab Pelidotin (Cofe‐P),^[^ ^25^ ^]^ a PTK7‐targeting antibody‐drug conjugate (**Figure** **2**a). We genetically fused a FLAG‐tagged scFv_(PTK7)_ to the C‐terminus of scFv_(sortilin)_ and PGRN_493‐593_ using a short Gly‐Ser (GS) linker, and synthesized the corresponding (m1Ψ)‐modified mRNAs, designated SORT × PTK7 and PGRN × PTK7, through a time‐ and cost‐efficient manufacturing process (Figure 2b). The integrity and encapsulation efficiency of these mRNAs were evaluated via gel electrophoresis, capillary gel electrophoresis and transmission electron microscopy (Figure 2c,d; Figures S2 and S3, Supporting Information).

Subsequently, we evaluated the efficacy of these constructs in degrading PTK7. MDA‐MB‐468 cells were treated with gradient concentrations of PGRN × PTK7 and SORT × PTK7, and PTK7 levels were quantified by Western blot 48 h post‐treatment. Both constructs significantly promoted PTK7 degradation, achieving degradation rates of 79% and 94% at a concentration of 3 µg mL^−1^, respectively (Figure 2e). At a lower concentration of 1 µg mL^−1^, SORT × PTK7 induced substantial PTK7 degradation (≈90%) after 48 h, while PGRN × PTK7 resulted in ≈45% degradation. These results indicate that both scFv_(sortilin)_ and PGRN_493‐593_ can mediate the internalization and degradation of PTK7 via sortilin, with scFv_(sortilin)_ exhibiting greater targeting efficiency.

To further confirm that MedTAC technology mediates the degradation of MPs through binding to sortilin, we designed an mRNA encoding scFv_(PTK7)_ fused to a lysosome‐sorting sequence (PTK7 X CPP‐LSS, Figure 2c). Chen et al. demonstrated that covalently engineered nanobodies conjugated with CPP‐LSS can direct MPs to lysosomes for degradation through irreversible interactions.^[^ ^26^ ^]^ However, PTK7 X CPP‐LSS showed poor degradation efficiency, with a maximum degradation rate of only ≈29% (Figure 2e), indicating that the lack of specific binders for sortilin limited effective PTK7 degradation. Given its superior performance and orthogonality to native ligands, scFv_(sortilin)_ was selected as the optimal sortilin‐specific binder for further research. In summary, SORT X PTK7, hereafter referred to as MedTAC_PTK7_, effectively induces PTK7 degradation, positioning sortilin as a promising LTR candidate.

### 2.3 Sortilin‐Dependent Lysosomal Degradation of PTK7

**Figure 3:**
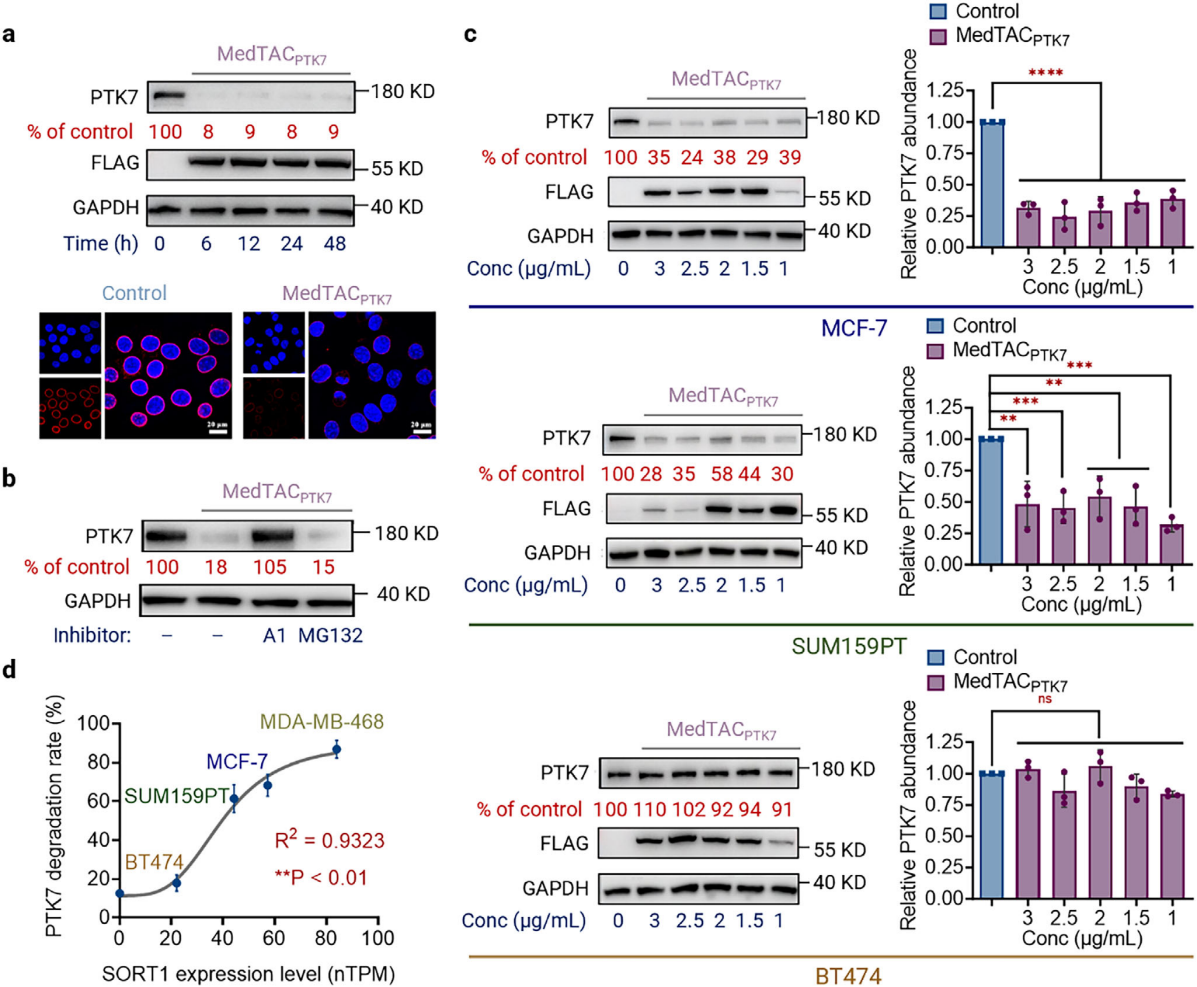
Kinetics and specificity of MedTAC‐induced degradation. a) Time‐course analysis demonstrated that PTK7 levels in MDA‐MB‐468 cells treated with 2 µg mL^−1^ MedTAC_PTK7_ significantly decreased as early as 6 h, with sustained degradation observed after 48 h. Representative immunofluorescence images of MDA‐MB‐468 cells after 48 h of treatment with 2 µg mL^−1^ MedTAC_PTK7_ or control (PTK7 in red; DAPI in blue). Scale bar: 20 µm. b) Pretreatment with 100 nM bafilomycin or 5 µM MG132 for 6 h, followed by 48 h of 2 µg mL^−1^ MedTAC_PTK7_ treatment, confirms lysosomal‐mediated PTK7 degradation. c) Western blot analysis shows total PTK7 levels across cancer cell lines (MCF‐7, SUM159PT, BT474) following 48 h of MedTAC_PTK7_ treatment. Quantitative data are presented as mean ± SD (n = 3). d) Summary of mean PTK7 degradation across different cell lines following 48 h of 1 µg mL^−1^ MedTAC_PTK7_ treatment, presented as mean ± SEM from three biologically independent experiments. All the p values were determined by ordinary one‐way ANOVA. n.s., not significant; ****^p^ < 0.0001, ***^p^ < 0.001, **^p^ < 0.01.

We further characterized the MedTAC‐mediated internalization and degradation. Time‐course analysis showed that MedTAC_PTK7_ rapidly and consistently reduced PTK7 levels in MDA‐MB‐468 cells, achieving a ≈92% reduction within 6 h of treatment with 2 µg mL^−1^ (**Figure** **3**a). After 48 h, degradation efficiency remained high at ≈91%. Confocal fluorescence imaging confirmed the near‐complete PTK7 removal after 48 h, highlighting the strong efficacy of MedTACs in protein degradation (Figure 3a). To determine whether degradation occurred via lysosomal or proteasomal pathways, MDA‐MB‐468 cells were pretreated with bafilomycin A1 (a lysosomal acidification inhibitor) or MG132 (a proteasome inhibitor) for 6 h, followed by MedTAC_PTK7_ treatment for 48 h. Bafilomycin A1 significantly mitigated PTK7 degradation, whereas MG132 had no effect, confirming that MedTACs promote lysosomal degradation (Figure 3b). These results indicate that MedTAC_PTK7_ induces rapid and sustained lysosomal degradation of PTK7.

Given the cell‐specific expression of sortilin, we hypothesized that MedTAC could facilitate protein degradation in a sortilin‐dependent manner. Treatment of MDA‐MB‐468 cells with LNP‐encapsulated mRNA‐encoding scFv_(PTK7)_ for 48 h did not result in a significant reduction in PTK7, suggesting that effective degradation requires the presence of sortilin on the cell surface (Figure S4, Supporting Information). We further evaluated PTK7 degradation in three additional cancer cell lines (MCF‐7, SUM159PT and BT474), characterized by varying sortilin expression but stable PTK7 levels, as confirmed by the HPA database. After 48 h of MedTAC_PTK7_ treatment, substantial PTK7 degradation was observed in MCF‐7 and SUM159PT cells, with mean degradation rates of 68% and 61%, respectively (Figure 3c). In contrast, BT474 cells showed minimal PTK7 degradation (22.1%), slightly higher than the 13% reduction seen with mRNA‐encoding scFv_(PTK7)_ treatment group (Figure 3c). Immunofluorescence microscopy further confirmed marked PTK7 reduction on the cell surface in MCF‐7 and SUM159PT, while BT474 showed no significant change (Figure S5, Supporting Information). These results suggest that the efficiency of PTK7 degradation positively correlates with sortilin expression levels (Figure 3d). This finding is consistent with those of Han et al., who reported that the efficient degradation of c‐Met and PTK7 by ITACs required moderate levels of IGF2R surface expression.^[^ ^27^ ^]^ Overall, our study demonstrates that MedTAC induce lysosome‐mediated degradation of PTK7 and the degradation efficiency is directly dependent on sortilin expression levels.

### 2.4 Expanding to Other Therapeutically Relevant MPs

**Figure 4:**
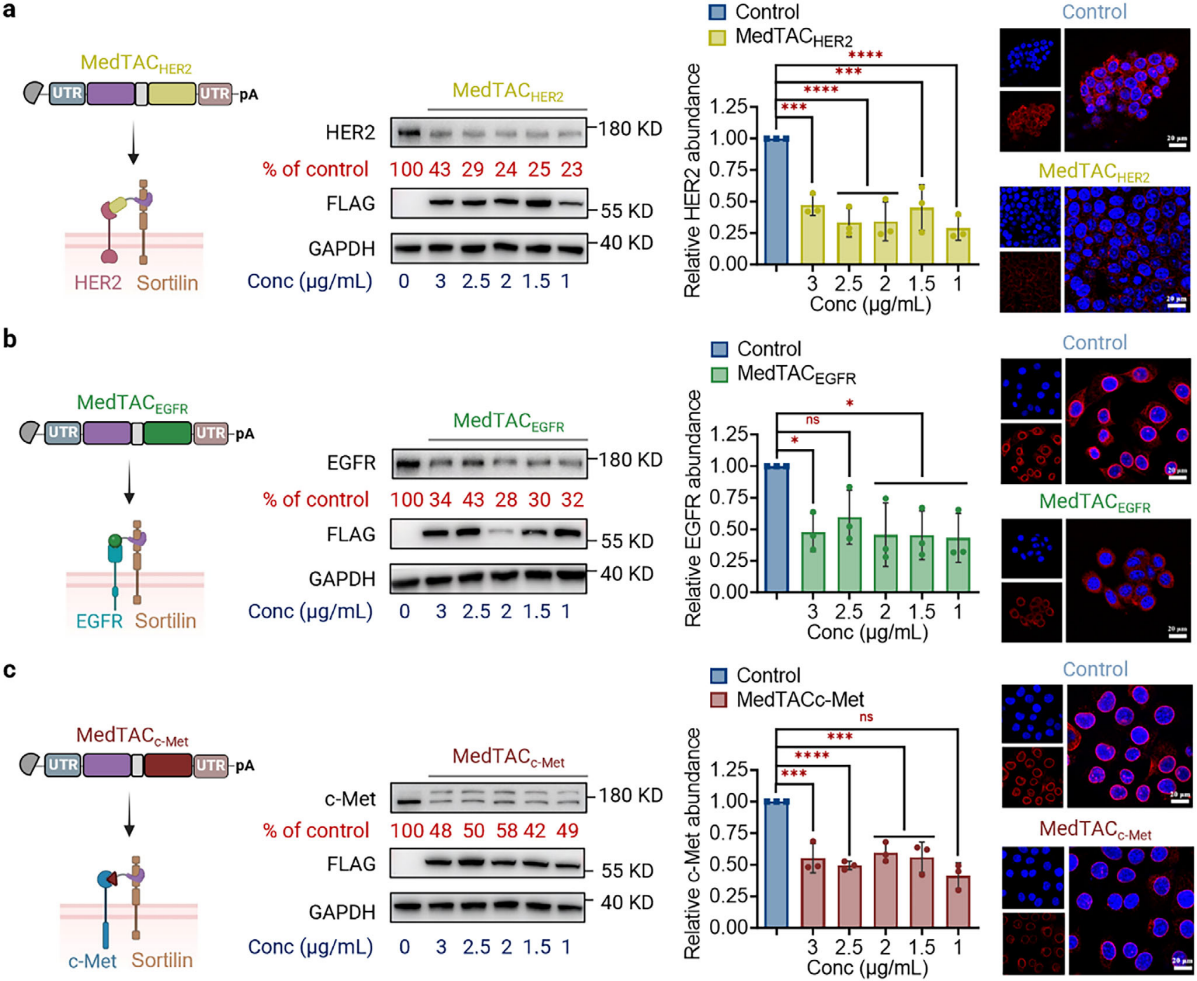
Targeted degradation of various therapeutically relevant MPs. a) Western blot analysis and representative immunofluorescence images of HER2‐expressing MCF‐7 cells treated with MedTAC_HER2_ for 48 h. HER2 appears in red and DAPI‐stained nuclei appears in blue. Scale bar: 20 µm. Data are presented as mean ± SD (n = 3). b) Western blot analysis and representative immunofluorescence images of EGFR‐expressing SK‐BR‐3 cells after 48‐h MedTAC_EGFR_ treatment. EGFR is shown in red, with DAPI in blue. Scale bar: 20 µm. Data are presented as mean ± SD (n = 3). c) Western blot and representative immunofluorescence images of c‐Met‐expressing MDA‐MB‐468 cells treated with MedTAC_c‐Met_ for 48 h. c‐Met is in red, DAPI is in blue. Scale bar: 20 µm. Data are presented as mean ± SD (n = 3). All the *p* values were determined by ordinary one‐way ANOVA. n.s., not significant; ****^p^ < 0.0001; ***^p^ < 0.001; *^p^ < 0.05.

To explore the versatility of the MedTACs platform, we investigated its potential to target additional therapeutically relevant MPs. We initially focused on human epidermal growth factor receptor 2 (HER2), which is commonly overexpressed in cancer cells and closely associated with tumor invasion and progression.^[^ ^28^ ^]^ We engineered MedTAC_HER2_ by fusing an engineered scFv_(HER2)_ derived from trastuzumab (Herceptin)^[^ ^29^ ^]^ with scFv_(sortilin)_ (Figure S6a, Supporting Information). After 48 h of treatment, significant HER2 degradation was observed in HER2‐expressing MCF7 and MDA‐MB‐231 cells, with degradation efficiencies of 77% and 72%, respectively (**Figure** **4**a; Figure S7, Supporting Information). Interestingly, higher concentrations of MedTAC_HER2_ led to decreased degradation efficiency, possibly due to a “hook effect” at saturation.

Next, we targeted EGFR, known for its role in cell proliferation, anti‐apoptotic functions and metastatic.^[^ ^2^ ^]^ MedTAC_EGFR_ was developed by fusing an engineered scFv_(EGFR)_, derived from cetuximab (a clinically approved EGFR inhibitor),^[^ ^30^ ^]^ with scFv_(_sortilin_)_ (Figure S6b, Supporting Information). After 48 h of MedTAC_EGFR_ treatment, SK‐BR‐3 cells showed a significant 72% reduction in EGFR levels (Figure 4b).

Lastly, we assessed MedTAC's ability to degrade the hepatocyte growth factor receptor (c‐Met), a receptor involved in cancer cell proliferation, invasion and metastasis.^[^ ^31^ ^]^ MedTAC_c‐Met_ was created by substituting the scFv_(PTK7)_ with an scFv derived from telisotuzumab^[^ ^32^ ^]^ to specifically target c‐Met (Figure S6c, Supporting Information). Treatment of MDA‐MB‐468 cells with MedTAC_c‐Met_ for 48 h resulted in a 58% reduction in c‐Met levels compared to controls (Figure 4c). Collectively, these findings highlight the modularity and versatility of MedTAC for targeting various therapeutically relevant MPs, underscoring its broad applicability as a versatile tool in precision cancer therapy.

### 2.5 Targeting Invasion and Migration in Triple‐Negative Breast Cancer (TNBC)

Breast cancer (BC) is the most prevalent cancer among women and the leading cause of cancer‐related mortality worldwide, with ≈2.3 million new cases and over 600000 deaths annually.^[^ ^33^ ^]^ TNBC, a highly invasive BC subtype, accounts for ≈12% to 17% of all cases. Compared to other BC subtypes, TNBC has higher relapse and metastasis rates, leading to poorer prognoses and survival outcomes.^[^ ^34^ ^]^ The lack of effective targeted therapies contributes to particularly poor clinical outcomes for patients with metastatic TNBC.

**Figure 5:**
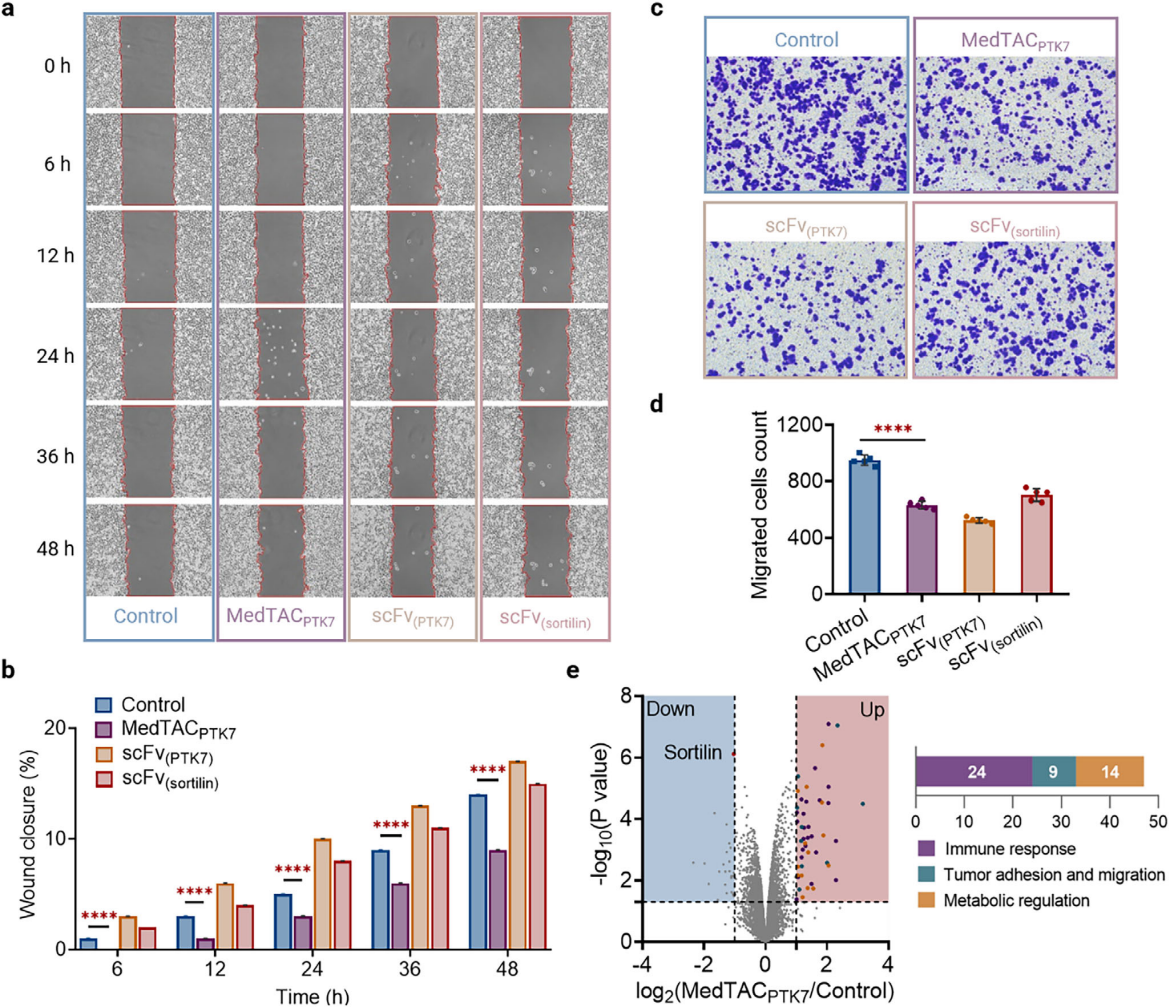
MedTAC_PTK7_ treatment reduces invasion and migration of TNBC cells. a) Representative light microscopy images from wound healing assays of MDA‐MB‐468 cells, showing migration rates at various time points following treatment with MedTAC_PTK7_, mRNA‐encoding scFv(_PTK7_), and mRNA‐encoding scFv(_sortilin_). Scale bar: 200 µm. b) Data of wound healing assay data presented as mean ± SD, n = 3 per group. Statistical significance was determined by two‐way ANOVA; ****^p^ < 0.0001. c) Transwell assay of MDA‐MB‐468 cells following treatment with MedTAC_PTK7_, mRNA‐encoding scFv(_PTK7_), and mRNA–encoding scFv(_sortilin_). Scale bar, 200 µm. d) Data of transwell assay are presented as mean ± SD, n = 5 per group. Statistical significance was determined by t‐test; ****^p^ < 0.0001. e) Volcano plot depicting differentially expressed proteins in MDA‐MB‐468 cells treated with MedTAC_PTK7_ for 48 h relative to untreated cells. Forty‐seven significantly upregulated proteins, primarily related to immune response pathways, tumor adhesion and migration, and metabolic regulation, are highlighted [log2(MedTAC_PTK7_/Control) ≥ 1, *p* < 0.05, n = 3].

PTK7, a catalytically inactive receptor tyrosine kinase, is significantly enriched in tumor‐initiating cells in TNBC, although its specific oncogenic roles remain elusive.^[^ ^35^ ^]^ To elucidate PTK7's role in TNBC cell invasion, migration and proliferation, we utilized MedTAC_PTK7_ to selectively degrade PTK7 in MDA‐MB‐468 cells. Wound healing assays showed a significant decrease in cell migration after 48 h of treatment with MedTAC_PTK7_ compared to controls, corroborating previous findings^[^ ^36^ ^]^ (**Figure** **5**a,b). Interestingly, targeting the extracellular domains of PTK7 and sortilin separately using mRNA‐encoding scFv_(PTK7)_ and mRNA‐encoding scFv_(sortilin)_ led to increased cellular motility. This suggests that PTK7's functions extend beyond the extracellular region to the intracellular region, indicating that complete PTK7 degradation may more effectively inhibit TNBC cell migration. Furthermore, transwell assays demonstrated a marked reduction in invasiveness across treatment groups, likely due to the disruption of PTK7‐sortilin interactions, which impairs basement membrane reconstruction and thus inhibits cell invasion (Figure 5c,d). The anti‐proliferative effects of MedTAC_PTK7_ were also evaluated using the Cell Counting Kit‐8 (CCK8) assay, revealing no significant cytotoxicity in MDA‐MB‐468 cells, even at concentrations up to 4 µg mL^−1^ (Figure S8, Supporting Information). Collectively, these results indicate that MedTAC_PTK7_ can selectively and effectively target PTK7‐positive TNBC cells in vitro, mainly by reducing cell invasion and migration.

To further explore the proteome‐wide changes induced by MedTAC_PTK7_, we performed data‐independent acquisition (DIA) mass spectrometry (Figure 5e). MDA‐MB‐468 cells were treated with MedTAC_PTK7_ for 48 h, followed by whole‐cell lysates analysis. Notably, PTK7 levels showed minimal change compared to controls, whereas sortilin degradation was significant. This differential effect could be attributed to the distinct subcellular localizations of these proteins with PTK7 primarily localized at the plasma membrane and sortilin across various compartments, including the cytosol. Low PTK7 detectability in whole‐cell lysates might reflect its low abundance, as previously reported.^[^ ^6^ ^]^ Proteomic analysis identified 47 significantly upregulated proteins following MedTAC_PTK7_ treatment. Functional enrichment analysis revealed that 51% of these proteins were associated with immune response pathways, 19% with tumor cell adhesion and migration, and 30% with metabolic regulation. These findings suggest that MedTAC_PTK7_ affects broader cellular processes beyond inhibiting TNBC invasion and migration. The significant upregulation of immune‐related proteins implies a potential role for MedTAC_PTK7_ in modulating the tumor immune microenvironment, warranting further investigation through in vivo studies and functional assays.

### 2.6 MedTAC‐Mediated Long‐Term Tumor Suppression In Vivo

**Figure 6:**
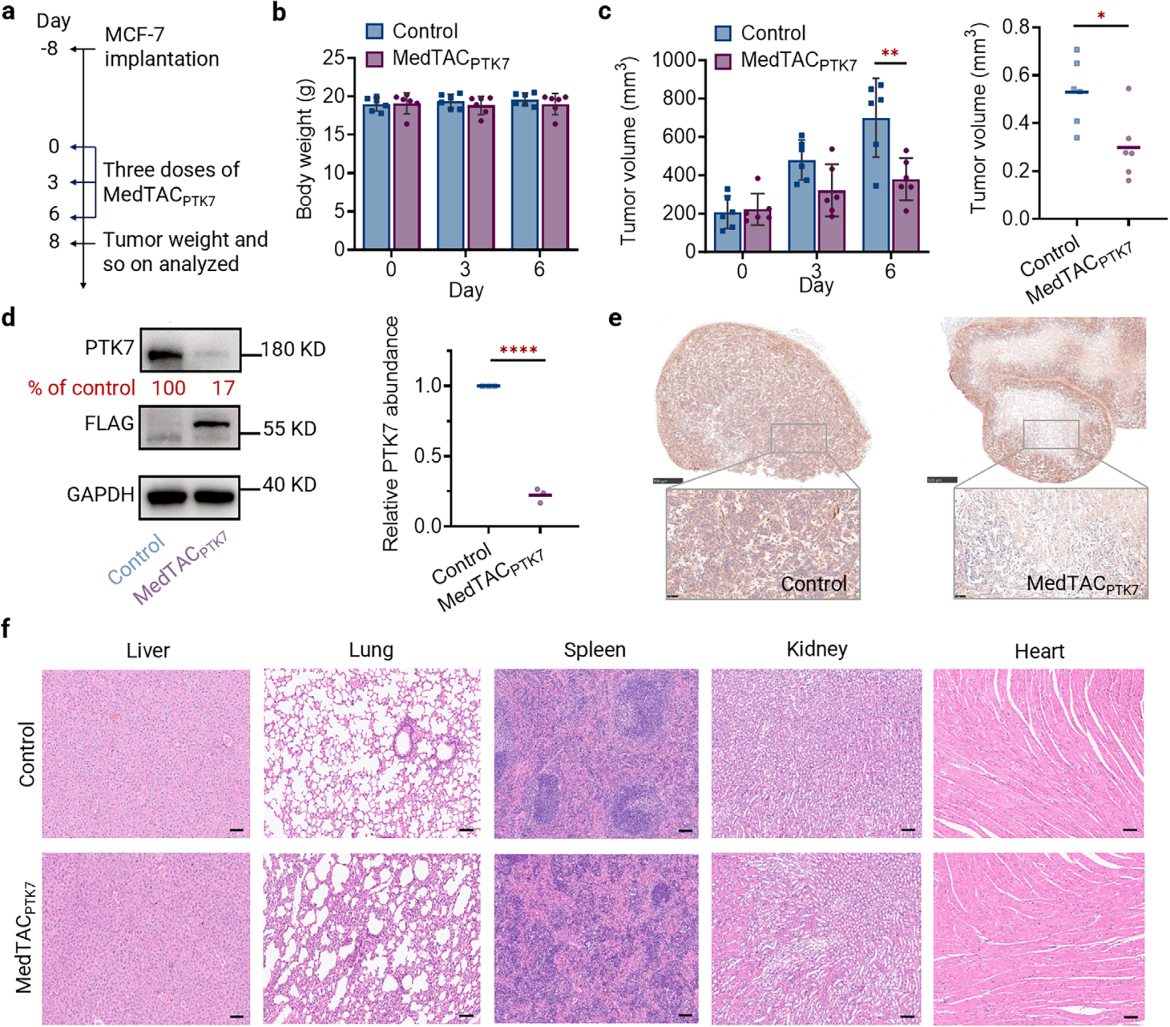
MedTAC_PTK7_ treatment suppresses tumor growth in vivo. a) Experimental design for assessing the therapeutic efficacy of MedTAC_PTK7_ in tumor‐bearing mice. b) Weight measurements following MedTAC_PTK7_ treatment showing no toxicities (n = 6 per group). c) Average tumor volume and weight of mice post‐treatment. Error bars represent mean ± SD, n = 6 mice per group. Statistical significance was determined by t test; **^p^ < 0.01, *^p^ < 0.05. d) PTK7 expression in tumor tissue quantified by Western blot after three treatment doses. e) Quantification of PTK7 expression in tumor sections via IHC. Scale bars: 500 µm (4× magnification) and 50 µm (20× magnification). f) H&E staining of main organs after 3‐dose treatment with DPBS or MedTAC_PTK7_. Scale bars: 50 µm (20× magnification).

Given the promising efficacy of MedTACs, we conducted an in‐depth evaluation of MedTAC_PTK7_’s therapeutic potential, pharmacokinetics and safety in BALB/c nude mouse models. MCF‐7‐bearing mice were randomly assigned to receive intra‐tumoral injections of either DPBS or LNP‐MedTAC_PTK7_. Once tumor volumes reached ≈200 mm^3^, treatments (0.5 mg kg^−1^) were administered every three days (**Figure** **6**a). After three doses, no statistically significant changes in body weight were observed, indicating no acute systemic toxicity (Figure 6b). Upon treatment completion, tumors were excised, weighed, and analyzed via immunohistochemistry (IHC) and hematoxylin‐eosin (H&E) staining. LNP‐MedTAC_PTK7_ significantly inhibited tumor growth, reducing tumor volume and weight by over 40% compared to controls (Figure 6c; Figure S9, Supporting Information). Consistent with Western blot data, PTK7 levels were substantially reduced in the tumor core but showed minimal change at the margins (Figure 6d,e). H&E staining of major organs (liver, lungs, spleen, kidneys and heart) revealed no systemic toxicity from LNP‐MedTAC_PTK7_ treatment (Figure 6f).

**Figure 7:**
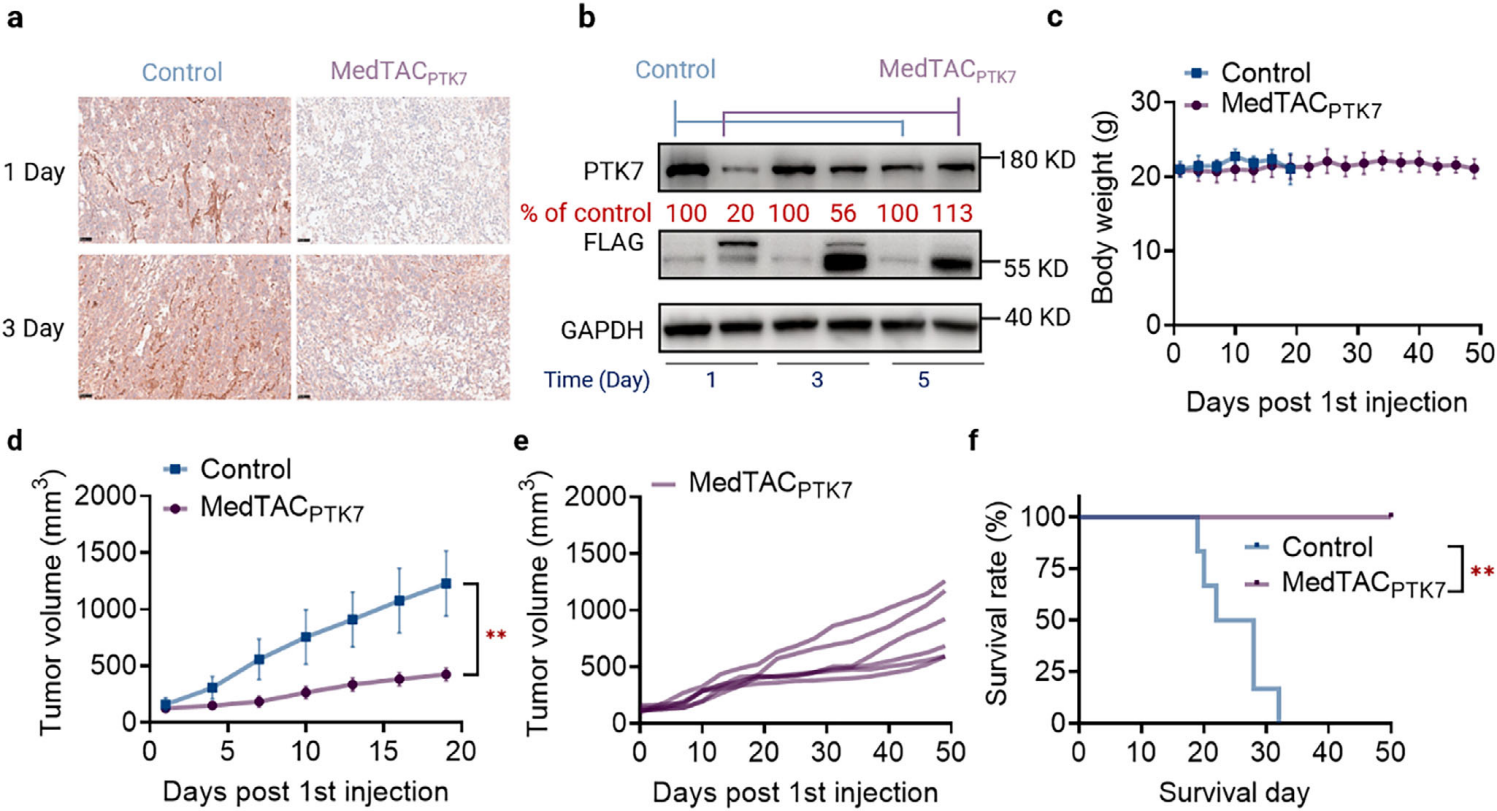
MedTAC_PTK7_ induces sustained PTK7 degradation in vivo and extends mouse survival. a) Quantification of PTK7 expression in tumor sections at 1 day and 3 days post‐MedTAC_PTK7_ treatment, as assessed by IHC. Scale bars: 50 µm (20× magnification). b) Quantification of PTK7 expression in tumor tissues at 1 day 3 days and 5 days post‐MedTAC_PTK7_ treatment, as assessed by Western blot. c) Body weight measurements of mice during treatment (n = 6 per group). d) Average tumor of mice post‐treatment. Error bars represent mean ± SD (n = 6 mice per group). Statistical significance was determined by t test; **^p^ < 0.01. e) Changes in average tumor volume following MedTAC_PTK7_ treatment or control administered every three days over a period of 50 days. f) Survival of mice treated with MedTAC_PTK7_ or control over a period of 50 days. Statistical significance was determined by t test; **^p^ < 0.01.

Lysosome‐targeting degradation with a prolonged pharmacokinetic profile represents a promising therapeutic approach, allowing sustained yet reversible protein degradation. Previous research demonstrated that low‐dose, chemically modified mRNA could sustain in vivo bispecific scFv production for almost a week.^[^ ^15c^ ^]^ Here, we explored whether MedTAC could achieve similar long‐term pharmacokinetics in vivo. Tumor‐bearing mice received a single 0.5 mg kg^−1^ dose of MedTAC_PTK7_, which effectively reduced PTK7 levels in the tumor core, maintained for at least three days (**Figure** **7**a). Western blot analysis confirmed an 80% reduction in PTK7 levels one day post‐treatment, with a sustained 44% degradation three days later, indicating favorable pharmacokinetics. Additionally, membrane protein degraders were detectable up to day 3, but by day 5, their expression levels were nearly undetectable, suggesting clearance within this timeframe (Figure 7b). Single‐dose treatment significantly inhibited tumor growth compared to controls (Figure S10, Supporting Information). H&E staining further confirmed that MedTAC_PTK7_ was safe, with no adverse effects on major organs observed at 1 and 3 days post‐administration (Figure S11, Supporting Information).

Given its robust pharmacodynamic effects, we evaluated MedTAC_PTK7_’s impact on survival in MCF‐7 xenograft mouse models. Once tumor volumes reached ≈125 mm^3^, treatments were administered every three days. Throughout the treatment, MedTAC_PTK7_ significantly suppressed tumor growth without noticeable body weight loss (Figure 7c,d). By day 18, mortality was observed in the control group, while all MedTAC_PTK7_‐treated mice remained alive. Although MedTAC_PTK7_ treatment significantly extended mouse survival to over 50 days, compared to 20–30 days for controls, treated mice still showed a time‐dependent increase in tumor volume (Figure 7e,f). These findings suggest that PTK7 degradation alone may not be sufficient to fully eliminate tumors, and combining MedTAC_PTK7_ with other therapies could further enhance its therapeutic efficacy. Overall, these results underscore the promising pharmacokinetic profiles, biosafety and tumor suppression effects of MedTAC_PTK7_, with PTK7 degradation contributing to extended survival in BC‐bearing mice.

## 3 Conclusion

Lysosome‐targeting degradation (LTD) has emerged as a promising strategy for removing dysregulated MPs, offering robust modulation of pathological cellular behavior. However, conventional LTD technologies face challenges, including limited availability of tissue‐ or cell‐specific LTRs, suboptimal pharmacokinetics and complex chemical synthesis. In this work, we sought to address these issues by introducing a novel MedTAC platform that enables the precise degradation of therapeutically relevant MPs via a sortilin‐dependent mechanism.

A significant highlight of the MedTAC technology is leveraging sortilin, a receptor known to mediate endocytosis and lysosomal trafficking. Bioinformatics analysis shows sortilin's predominant expression in the brain and various cancers, with minimal presence in other normal tissues. During manuscript review, Huang et al. reported that engineered EndoTags targeting sortilin could specifically degrade EGFR at the cellular level.^[^ ^13^ ^]^ Comparatively, our study presents further findings: 1) Sortilin facilitates the rapid and efficient degradation of multiple therapeutically relevant MPs, including PTK7, HER2, EGFR and c‐Met, as demonstrated in comprehensive in vitro and in vivo experiments; 2) Comparisons between native and engineered sortilin ligands underscore the importance of binders that avoid endogenous ligand binding sites. We observed a positive correlation between MedTAC‐mediated protein degradation efficiency and sortilin expression levels, supported by both quantitative and qualitative analyses. Additionally, MedTAC_PTK7_ was found to reduce the invasion and migration of TNBC cells and extend survival in BC‐bearing mouse with minimal systemic toxicity. This establishes sortilin as a promising candidate for LTRs, although the ongoing development of cell‐ or tissue‐specific LTRs remains critical to broaden the therapeutic window and enhance clinical applications. Another notable aspect of MedTAC is its innovative use of mRNA vectors to deliver bispecific degraders, addressing key challenges of traditional LTD technologies, such as the need for specialized expertise and labor‐intensive synthesis. Unlike conventional methods, MedTAC can be produced from plasmids within 3–5 days, similar to the rapid development seen with SARS‐CoV‐2 mRNA vaccines.^[^ ^37^ ^]^ This expedited process reduces turnaround times and enhances scalability, making MedTAC suitable for high‐demand and personalized treatments. One of its major strengths is its pharmacokinetic profile; a single low‐dose administration of MedTAC_PTK7_ sustained reduced PTK7 levels for over three days, suggesting prolonged in vivo activity. This could lead to less frequent dosing and improved patient compliance. Our findings align with BioNTech's previous report of mRNA‐encoded bispecific scFv retaining potent cell‐killing activity for up to 6 days in vivo.^[^ ^15c^ ^]^ Collectively, these advantages highlight the potential of MedTAC as a next‐generation therapeutic, expanding the therapeutic landscape for conditions previously difficult to target.

In conclusion, this study introduces a novel LTD technology that offers a scalable, rapid‐response and cell‐specific approach to degrading cancer‐related MPs. Nonetheless, challenges persist, such as the need for ligands with higher affinity and specificity for effective MP recognition, as well as optimizing LNPs for precise tumor targeting to minimize off‐target effects. Future efforts will focus on improving ligand design through advanced screening technologies, including phage display and in silico protein modeling, enhancing tissue‐specific delivery via customized LNPs,^[^ ^38^ ^]^ and exploring MedTAC's potential to address resistance mutations and previously undruggable proteins. Additionally, combining MedTAC with immunotherapies could further enhance cancer treatment outcomes, paving the way for more effective and personalized therapeutic strategies.

## 4 Experimental Section

### Antibodies and Reagents

Anti‐PTK7 polyclonal antibody (catalog no. 25618, 1:1000), HER2 polyclonal antibody (catalog no. 41798, 1:1000), EGFR polyclonal antibody (catalog no. 51071‐2‐AP, 1:1000), c‐Met polyclonal antibody (catalog no. 8198, 1:1000), GAPDH polyclonal antibody (catalog no. 10494‐1‐AP, 1:2000), FLAG‐tag polyclonal antibody (catalog no. 66008‐4‐Ig, 1:2000), and beta‐actin polyclonal antibody (catalog no. 81115‐1‐RR, 1:2000) were purchased from commercial suppliers. Secondary polyclonal antibodies for Western blot included HRP‐conjugated affinipure goat anti‐mouse IgG (H+L) (catalog no. SA00001‐1, 1:2000) and HRP‐linked goat anti‐rabbit IgG (catalog no. 7074S, 1:2000). Small‐molecule inhibitors bafilomycin A1 (catalog no. SML1661) and MG132 (catalog no. M7449) were purchased from Sigma‐Aldrich.

### Cell Culture

Human breast cancer cell lines MDA‐MB‐468 and MDA‐MB‐231 were sourced from the American Type Culture Collection (ATCC) and cultured in Leibovitz's L‐15 medium supplemented with 10% fetal bovine serum (FBS, Invitrogen). BT‐474 cells, also sourced from ATCC, were maintained in RPMI‐1640 medium (Gibco) with 10% FBS. MCF‐7, SK‐BR‐3 and SUM159PT cells were cultured in DMEM (Gibco) supplemented with 10% FBS. All cell lines were incubated at 37 °C in a humidified atmosphere containing 5% CO_2_, except for the MDA‐MB 468 cell, which was maintained in a CO_2_‐free incubator.

### Animal Studies

Female BALB/c nude mice were obtained from Shanghai Slaccas Experimental Animal Co., Ltd. (Shanghai, China). Age‐ and sex‐matched animals were used for all experiments. Mice were maintained under specific pathogen‐free (SPF) conditions at the BioNTech SE animal facility in Mainz. All animal experiments received approval from the Animal Ethics Committee of the Hangzhou Institute of Medicine, Chinese Academy of Sciences (AP2024‐09‐0203).

### Plasmid Construction

All related expression plasmids were constructed by inserting human codon‐optimized DNA sequences encoding the target proteins into the pET‐28a (+) vector. These plasmids were synthesized by GenScript Biotech Corporation (China).

Cofetuzumab pelidotin (ABBV‐647/PF‐06647020) was an antibody‐drug conjugate (ADC) targeting PTK7, consisting of the PTK7‐targeting humanized monoclonal antibody cofetuzumab, a cleavable valine‐citrulline linker, and the microtubule inhibitor Aur0101. The anti‐PTK7 scFv was derived from the variable heavy (VH) and light (VL) sequences of cofetuzumab. Latozinemab (AL001) was an anti‐sortilin monoclonal antibody developed by Alector (NASDAQ: ALEC) designed to increase PGRN levels to treat neurodegenerative diseases. Therefore, the anti‐sortilin scFv was derived from the VH and VL sequences of AL001. Another binder targeting sortilin was derived from progranulin (PRGN). Additionally, it has been reported that a conjugated cell‐penetrating peptide (CPP) and lysosome‐targeting sequence (LSS) were incorporated to enhance the internalization and lysosomal degradation of the PTK7 scFv.

The anti‐HER2 scFv was based on a humanized variant of trastuzumab (Herceptin), an FDA‐approved antibody for HER2. The EGFR‐specific scFv sequences were synthesized based on the amino acid sequences of cetuximab. The VH and VL sequences for the anti‐c‐Met scFv were derived from telisotuzumab, a highly specific monoclonal antibody against c‐Met. The variable domains of the scFv constructs were linked by 15‐ to 25‐residue glycine‐serine (GS) linkers, and the anti‐sortilin and anti‐PTK7/HER2/EGFR/c‐Met scFvs were connected via six‐residue GS linkers. All bispecific scFv constructs included a secretion signal and a C‐terminal 1×FLAG tag. The nucleotide sequences for all bispecific scFv constructs are provided in the supplementary materials (Table S3, Supporting Information).

To ensure the accuracy of the design, it carefully analyzed the structural properties of the heavy and light chains of the above‐mentioned antibodies to guide the synthesis of scFv with high binding affinity and specificity to the target proteins.

### Plasmid Amplification and Purification

Plasmids encoding human codon‐optimized DNA sequences were constructed and amplified in E. coli BL21 using standard protocols. Below was the procedure for plasmid amplification and purification. Briefly, a 50 µL aliquot of *E. coli* BL21 culture, containing the target plasmid, was inoculated into 10 mL of Luria‐Bertani (LB) medium supplemented with 100 µg mL^−1^ kanamycin. The culture was incubated overnight at 37 °C with continuous shaking at 220 rpm to facilitate optimal bacterial growth. Following overnight incubation, the culture was expanded by transferring it to 100 mL of fresh LB medium, also containing 100 µg mL^−1^ kanamycin, to ensure robust plasmid production. The expanded culture was incubated under identical conditions for at least 12 h to achieve sufficient cell density.

After reaching the appropriate cell density, the bacterial culture was centrifuged at 8000 rpm for 5–10 min, and the supernatant was discarded. The cell pellet was retained for downstream plasmid extraction. Plasmid extraction was performed using the Endofree Maxi Plasmid Kit (TIANGEN, catalog no. DP117) based on the manufacturer's guidelines, with additional optimizations to improve yield. The bacterial pellet was initially resuspended in 5 mL of solution P1 to ensure complete dispersion. To lyse the cells, 5 mL of solution P2 was added, and the mixture was gently inverted 4–6 times, followed by incubation at room temperature for 1–2 min to allow complete lysis. Next, 7 mL of solution P3 was added to neutralize the lysate, forming a white precipitate. The mixture was centrifuged at 12000–14000 rpm for 10 min at room temperature, and the resulting clear supernatant was collected. The collected supernatant was carefully transferred to a new 50 mL centrifuge tube. An amount of isopropanol equal to 0.7 times the volume of the supernatant was added to precipitate the plasmid DNA, and the mixture was applied to a plasmid purification column. Following a 2‐min centrifugation, the purification column was placed in a new collection tube and allowed to air dry under sterile conditions in a biosafety cabinet for 2 min to remove any residual alcohol. The plasmid DNA was eluted by adding 1 mL of nuclease‐free water to the column. The quality and integrity of the eluted plasmids were validated by agarose gel electrophoresis, confirming the expected size and integrity. For precise quantification, a NanoDrop spectrophotometer (Thermo Fisher Scientific) was employed.

### In Vitro Transcription and mRNA Purification

Plasmids were linearized using BspQ I (NEB, catalog no. R0712L) and subsequently used as DNA templates for in vitro transcription (IVT) with T7 RNA polymerase and a transcription kit (Vazyme, catalog no. TR101‐01). During the reaction, uridine triphosphate (UTP) was replaced with N1‐methylpseudouridine (m1Ψ, Synthgene) to enhance transcription efficiency and mRNA stability. The resulting m7G5′ppp5′G2´‐O‐Met‐capped (cap1) IVT mRNAs were generated using vaccinia virus guanylyltransferase and 2′‐O‐methyltransferase (Novoprotein).

After transcription, RNA was purified using RNA clean beads, and the results were verified via agarose gel electrophoresis and assessed for quality with an Agilent 5400 Bioanalyzer. The purified IVT mRNA was quantified using a NanoDrop spectrophotometer (Thermo Fisher). The purified IVT mRNA was stored at −20 °C to maintain its stability and activity for subsequent experimental applications.

### LNP Formulation

Lipid nanoparticles (LNPs) were prepared using an ethanol‐drop nanoprecipitation method, as previously established in the literature. The lipids were first dissolved in ethanol at the following molar ratios: 50:10:38.5:1.5 (ionizable lipid (SM102): distearoyl phosphatidylcholine (PC): cholesterol: polyethylene glycol lipid). This lipid mixture was then rapidly mixed with a 20 mM sodium citrate‐hydrochloric acid buffer containing mRNA in a 1:2 ratio (lipid to mRNA, based on ethanol volume) using a microfluidic mixer (Precision Nanosystems).

After mixing, the LNPs were concentrated using Amicon Ultra centrifugal filters (EMD Millipore) to remove excess ethanol and unencapsulated materials. The concentrated LNP suspension was then filtered through a 0.22 µm membrane to eliminate any aggregated particles. Finally, the purified LNPs were stored at 4 °C for short‐term use or characterized for their physicochemical properties before further applications.

### LNP Characterization

The particle size (number average diameter) and zeta potential were determined using the dynamic light scattering (DLS) method in a Zetasizer (Malvern, UK). The encapsulation efficiency of LNP mRNA was indirectly assessed using the Quant‐iT Ribogreen RNA assay (Thermo Fisher). Additionally, the particle morphology was observed using transmission electron microscopy (TEM) to evaluate its structural characteristics and uniformity.

### AlphaFold‐Multimer Prediction and Analysis

In silico screening was conducted using the AlphaFold‐Multimer program on the ColabFold platform, following previously established protocols.^[^ ^17^, ^39^ ^]^ Briefly, AlphaFold‐Multimer calculations were performed by aligning structural sequences and generating five independent, unrelaxed models. The models were evaluated based on their ranking, prediction confidence (pLDDT scores) and inter‐chain interface accuracy (ipTM scores). Further refinements were made using structural alignment and analysis tools to ensure accurate modeling of protein‐protein interactions, providing a comprehensive assessment of potential binding interfaces. Scores for all predicted structures can be found in Table S2 (Supporting Information).

### Western Blot Analysis

To assess PTK7 degradation, 1 × 10^6^ MDA‐MB‐468 cells were seeded in a 6‐well plate. The cells were treated with mRNA encoding three different FLAG‐tagged degraders (PRGN × PTK7, SORT × PTK7, and PTK7 × CPP‐LSS) at concentrations ranging from 0 to 3 µg mL^−1^ for 48 h. Cells were treated with 2 µg mL^−1^ MedTAC_PTK7_, and PTK7 degradation efficiency was analyzed at different time points (0 to 48 h) to study the degradation kinetics. To investigate the involvement of lysosomal and proteasomal pathways, cells were pretreated with 100 nM bafilomycin A1 or 1 µM MG132 for 6 h, followed by treatment with 2 µg mL^−1^ MedTAC_PTK7_ for 48 h. The adaptability of MedTAC was further explored by using degraders targeting other receptors (such as HER2, EGFR, and c‐Met) in MCF‐7, MDA‐MB‐231, SK‐BR‐3, and MDA‐MB‐468 cells. Additionally, the role of sortilin expression in PTK7 degradation was studied by treating MCF‐7, SUM159PT, and BT474 cells with MedTAC_PTK7_ at concentrations ranging from 0 to 3 µg mL^−1^ for 48 h.

After treatment, cells were washed three times with DPBS and lysed on ice in RIPA buffer containing a mixture of protease and phosphatase inhibitors for 30 min. The lysates were centrifuged, and the supernatants were collected. Protein concentrations were determined using a BCA assay kit (Beyotime, catalog no. P0012). Equal amounts of protein (30 µg) were separated by 4–20% SDS‐PAGE and then transferred to polyvinylidene fluoride (PVDF) membranes. The membranes were blocked with 5% skim milk (prepared in 1× TBST) for 2 h, followed by overnight incubation at 4 °C with primary antibodies (1:2000 dilution in 5% skim milk). After washing the membranes three times with 1× TBST, they were incubated with secondary antibodies (1:2000 dilution in 5% skim milk) for 1–2 h at room temperature and then washed three more times with 1× TBST. Enhanced chemiluminescence (ECL) reagent (Biosharp, Beijing) was used for signal detection, and images were acquired using the ImageQuant 800 (AMERSHAM). Grayscale analysis was performed using ImageJ software.

### Immunofluorescence Imaging

Cells were initially plated onto confocal dishes and allowed to adhere for 24 h. They were then treated with 500 µL of complete growth medium containing either 2 µg mL^−1^ MedTACs or appropriate controls for 48 h. Following treatment, cells were washed three times with DPBS and fixed in 4% paraformaldehyde (PFA) for 15 min at room temperature. After an additional three washes with DPBS, the cells were blocked with 3% bovine serum albumin (BSA) in DPBS for 45 min. Subsequently, the cells were incubated overnight with primary antibodies at 4 °C, followed by incubation with secondary antibodies at 37 °C for 90 min. After washing with DPBS, the nuclei were stained with 500 µL of DAPI for 15 min in the dark, followed by three additional washes with DPBS. Fluorescence images were captured using a Nikon rotary confocal microscope (CSU‐W1‐SoRa) with laser settings of 488‐nm for the blue laser and 639‐nm for the red laser.

### Wound Healing Assay

MDA‐MB‐468 cells (≈1 × 10^5^) were seeded in wells of a 6‐well plate containing cell culture inserts and incubated overnight. After incubation, the inserts were removed, and the wells were washed with DPBS. The cells were then treated with MedTAC_PTK7_, mRNA‐encoding scFv(_PTK7_) or mRNA‐encoding scFv(_sortilin_) in a medium containing 2% FBS for 48 h. Images of wound closure were captured at 0, 6, 12, 24, 36, and 48 h using a light microscope with a 10× objective lens and a digital camera. The wound closure area in each image was quantified using ImageJ software (NIH, Bethesda, MD, USA). All experiments were conducted in triplicate to ensure statistical reliability.

### Transwell Assay

Transwell assays were performed using chambers with 8 µm pores (Costar Corporation) pre‐coated with Matrigel (BD Biosciences) to mimic the extracellular matrix. MDA‐MB‐468 cells were serum‐starved for 12–24 h to synchronize cell activity before treatment. Cells (1 × 10^5^) were treated with or without MedTAC_PTK7_, mRNA‐encoding scFv(_PTK7_) or mRNA‐encoding scFv(_sortilin_) and seeded into the upper chambers. The lower chambers contained 700 µL of medium supplemented with 20% FBS, serving as a chemoattractant to promote migration. After 48 h of incubation, cells that migrated to the lower side of the membrane were fixed with 1% paraformaldehyde (PFA) and stained with 0.1% crystal violet. The number of migrated cells was quantified using ImageJ software (NIH, Bethesda, MD, USA). Each experiment was repeated in triplicate to ensure reproducibility and statistical accuracy.

### CCK8 Assay

MDA‐MB‐468 cells (≈1 × 10^4^ per well) were plated in a 96‐well plate with 100 µL of complete growth medium and incubated overnight at 37 °C to allow for cell adherence. The next day, the cells were treated with varying concentrations of MedTAC_PTK7_, mRNA‐encoding scFv(_PTK7_) or mRNA‐encoding scFv(_sortilin_). After 48 h of treatment, 10 µL of CCK‐8 solution was added to each well, and the cells were incubated for an additional 4 h to enable the formation of formazan crystals. The absorbance was measured at 450 nm using an automated microplate reader (Thermo Fisher), and the data were analyzed to evaluate cell proliferation and viability under different treatment conditions. All experiments were performed in triplicate to ensure statistical robustness.

### Data‐Independent Acquisition (DIA) Mass Spectrometry

MDA‐MB‐468 cells were cultured in 10 cm dishes and treated with MedTAC_PTK7_ once they reached 70–80% confluency. After 48 h of treatment, ≈1 × 10^6^ cells were washed three times with ice‐cold DPBS and harvested using pre‐cooled scrapers. The cells were then lysed in RIPA buffer containing protease and phosphatase inhibitors for 30 min on ice. Following lysis, protein samples were digested with trypsin to generate peptides, which were subsequently analyzed using liquid chromatography‐tandem mass spectrometry (LC‐MS/MS) in Data‐Independent Acquisition (DIA) mode by Hangzhou Cosmos Wisdom Biotech Co., Ltd. This approach allows for comprehensive profiling of protein expression and post‐translational modifications, contributing to a deeper understanding of the cellular response to MedTAC_PTK7_ treatment.

### In Vivo Efficacy of MedTAC_PTK7_

Approximately 1 × 10^7^ MCF‐7 cells were suspended in 100 µL of PBS and injected subcutaneously into the right flanks of 6‐week‐old female BALB/c nude mice. Once the tumor volumes reached ≈200 mm^3^, the mice were randomly assigned to two groups (6 mice per group): a control group receiving DPBS and a treatment group receiving MedTAC_PTK7_ (0.5 mg kg^−1^, administered intratumorally every 3 days for a total of three doses). Tumor growth was monitored every 3 days by measuring the tumor length (L) and width (W) using calipers, and tumor volume (TV) was calculated using the formula TV = 0.5 × L × W^2^. Following the third injection, the mice were sacrificed, and tumor samples were collected for analysis via Western blotting and immunohistochemistry (IHC) to assess PTK7 expression. Histological evaluation was performed by Hubei BIOSSCI Biotechnology Co., Ltd., providing insights into the therapeutic effects of MedTAC_PTK7_ on tumor progression.

### Pharmacokinetics of MedTAC_PTK7_

Pharmacokinetics were evaluated in MCF‐7 xenograft mice. BALB/c nude mice (6–8 weeks, 16–18 g, n = 3) were injected subcutaneously with 1 × 10^7^ MCF‐7 cells to establish tumors. Once tumors were established, the mice were treated with either DPBS (100 µL) or MedTAC_PTK7_ (100 µL, 0.5 mg kg^−1^) for 1, 3, or 5 days. Following treatment, the mice were sacrificed, and tumors along with major organs (heart, liver, spleen, lung, and kidney) were harvested. Tumor tissues were washed with DPBS and homogenized in RIPA buffer supplemented with 1 mM protease inhibitor (Beyotime). After centrifugation at 12000 rpm for 10 min at 4 °C, equal volumes of supernatant were subjected to Western blot analysis for PTK7 detection. Immunohistochemical (IHC) staining for PTK7 and hematoxylin and eosin (H&E) staining of major organs were conducted by Hubei BIOSSCI Biotechnology Co., Ltd. to facilitate comprehensive histological evaluation of the pharmacokinetic profile and tissue distribution of MedTAC_PTK7_.

### Software

Data were analyzed and visualized using Microsoft Excel and GraphPad Prism, along with specific software tailored for each experiment. DNA and protein sequences were analyzed with SnapGene (v4.24), while images were created using BioRender. AlphaFold‐Multimer predictions were conducted on ColabFold and visualized using ChimeraX.

### Statistical Analysis

All statistical analyses were conducted using GraphPad Prism 10. Data were obtained from at least three biological replicates and were presented as the mean ± standard deviation (s.d.). Statistical significance was determined as follows: P‐value < 0.05 was considered statistically significant, while n.s. denoted no significant difference.

## Conflict of Interest

The authors declare no conflict of interest.

## Supporting information



Supporting Information

## Data Availability

The data that support the findings of this study are available in the supplementary material of this article.
