# Supplementary material for: Sortilin‐Mediated Rapid, Precise and Sustained Degradation of Membrane Proteins via mRNA‐Encoded Lysosome‐Targeting Chimera
Source: Adv Sci (Weinh). 2025 Apr 30;12(25):2501222. doi: 10.1002/advs.202501222 (PMC12224941; doi:10.1002/advs.202501222)
Supplement: Supplementary file 1 — Supporting Information [file ADVS-12-2501222-s001.docx]

Supporting Information

**Sortilin-Mediated Rapid, Precise and Sustained Degradation of Membrane Proteins via mRNA-Encoded Lysosome-Targeting Chimera**

*Xin Chang, Xinyu Qiu, Xiaoning Tong, Shaoju Gan, Weicheng Yi, Sitao Xie, Xiangsheng Liu, Chao Zuo, * and Weihong Tan **

**Supplementary Figures**

**
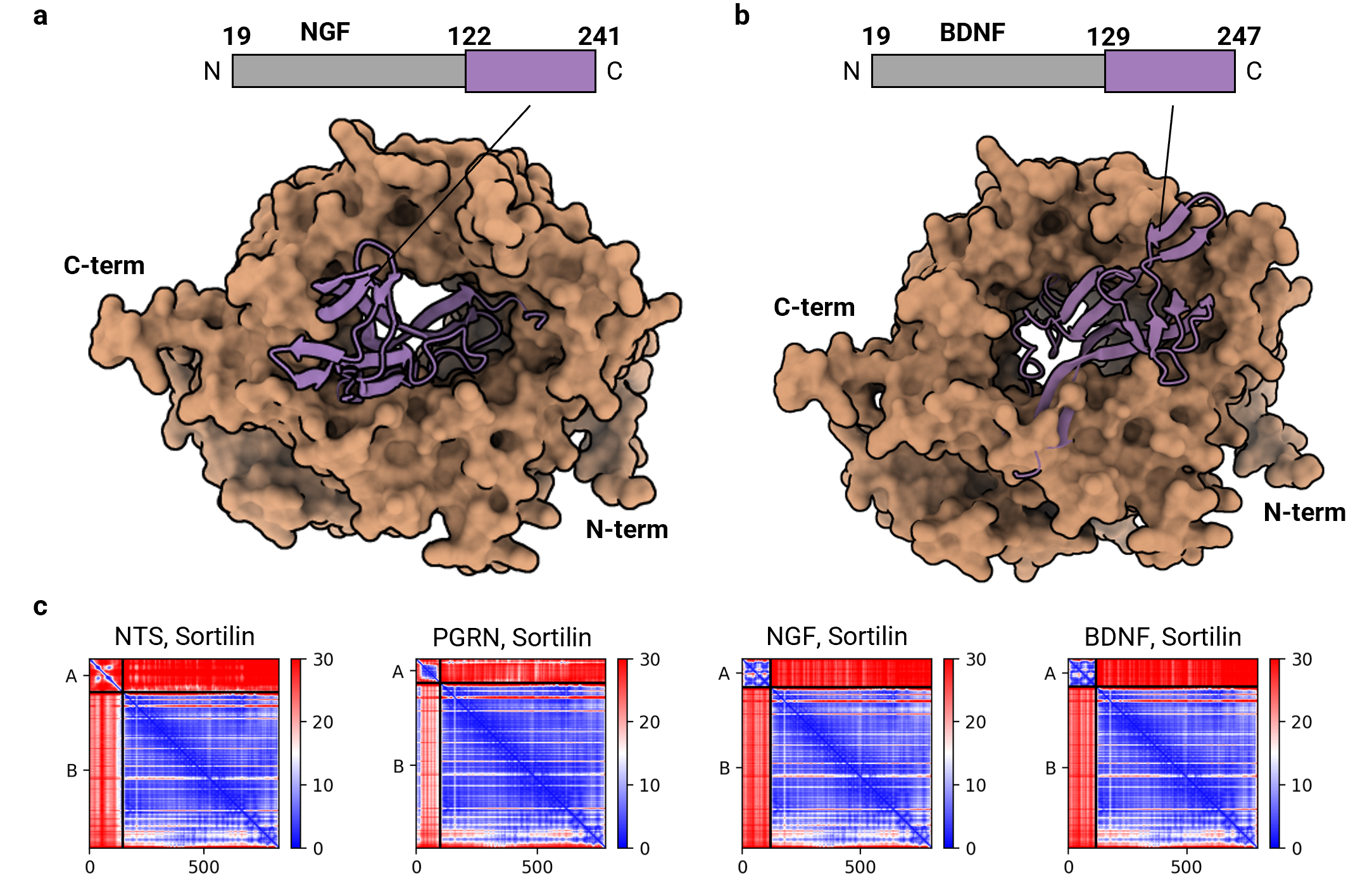
**

**Figure S1.** AlphaFold-Multimer predictions of the complex structures between endogenous ligands and sortilin. (a) Predicted structure of the NGF_122-241_/sortilin, with sortilin in light brown and NGF in purple brown. (b) Predicted structure of the BDNF_129-247_/sortilin, with sortilin in light brown and BDNF in purple brown. (c) Predicted aligned error (pAE) matrix for NTS, PGRN, NGF, and BDNF in complex with Sortilin.


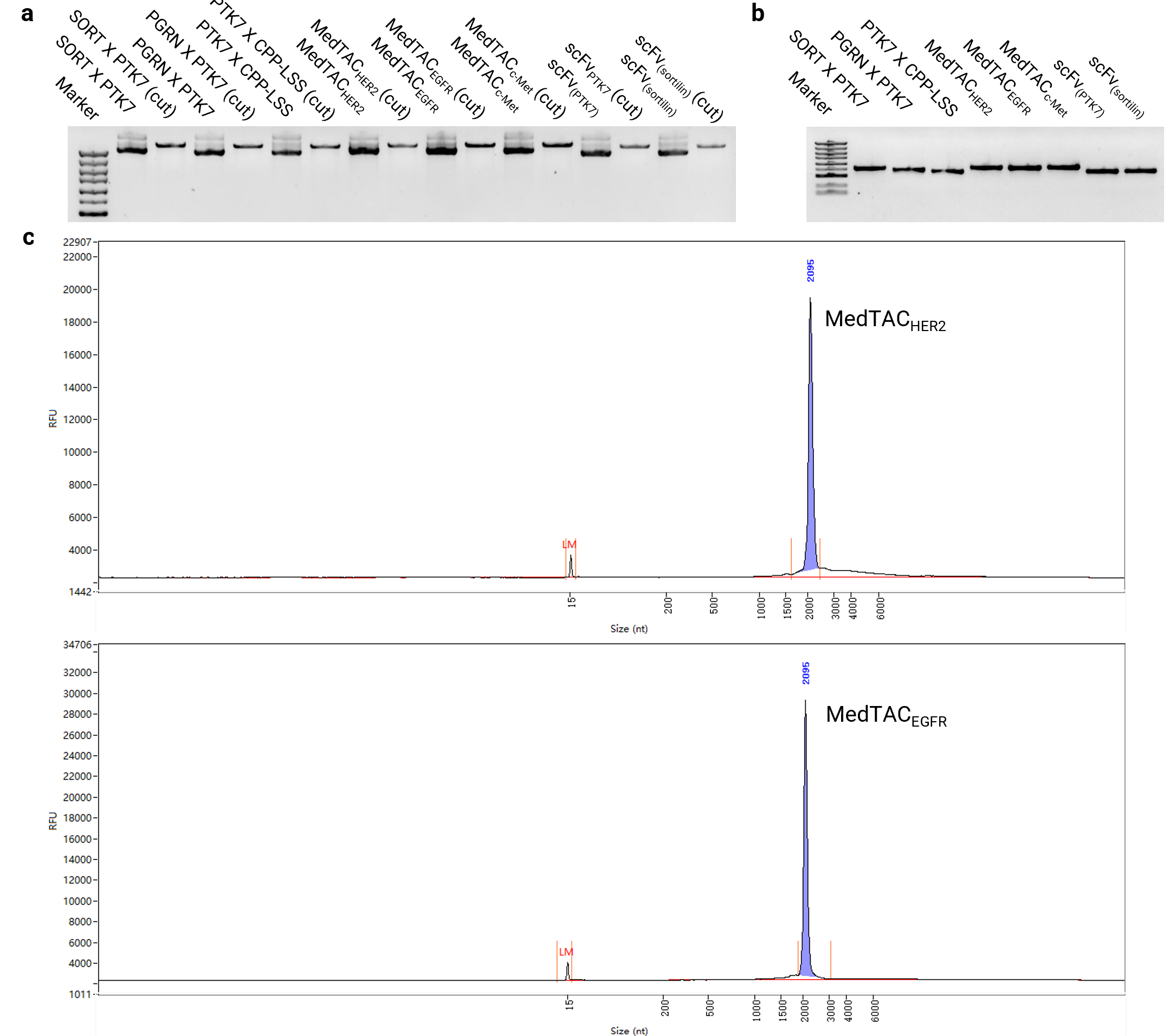


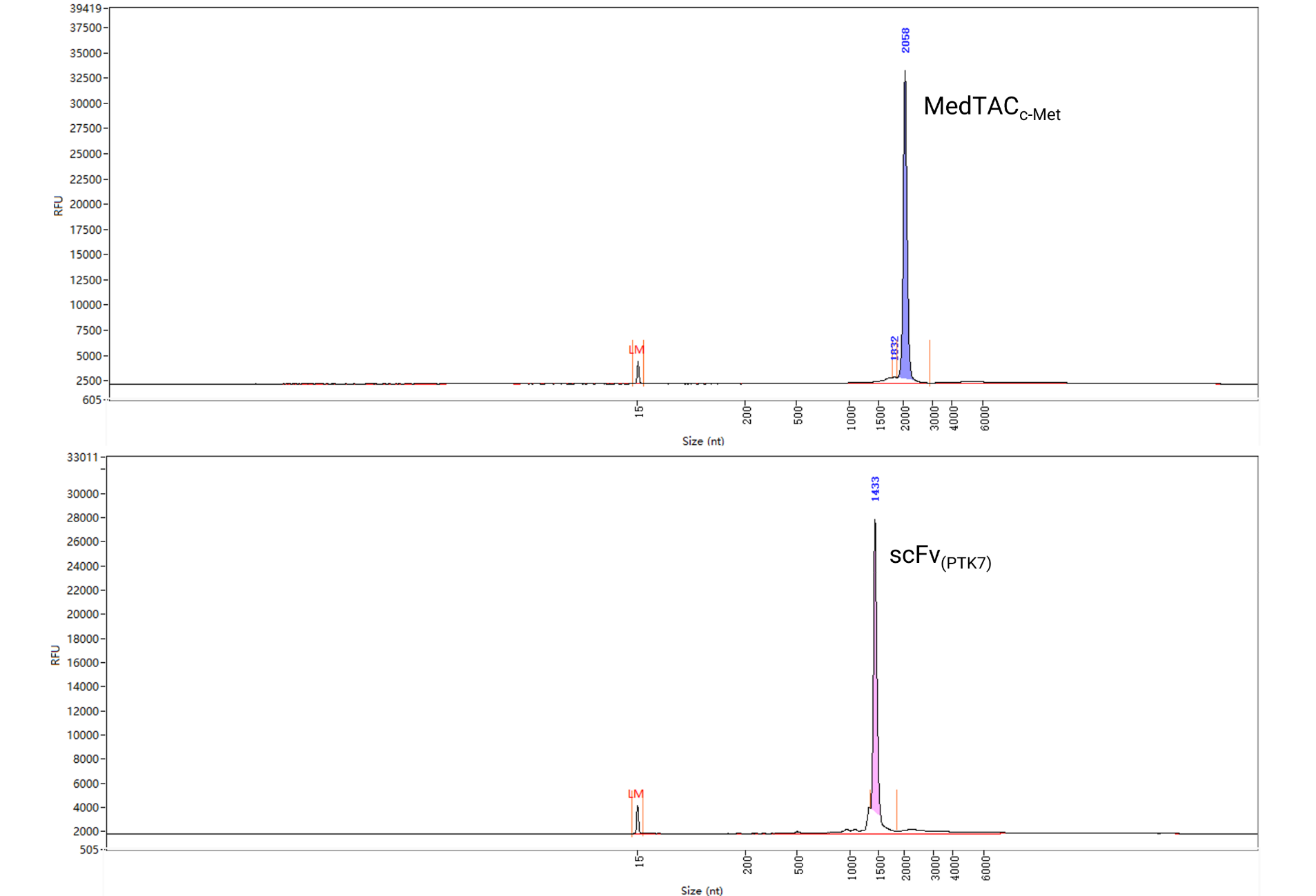


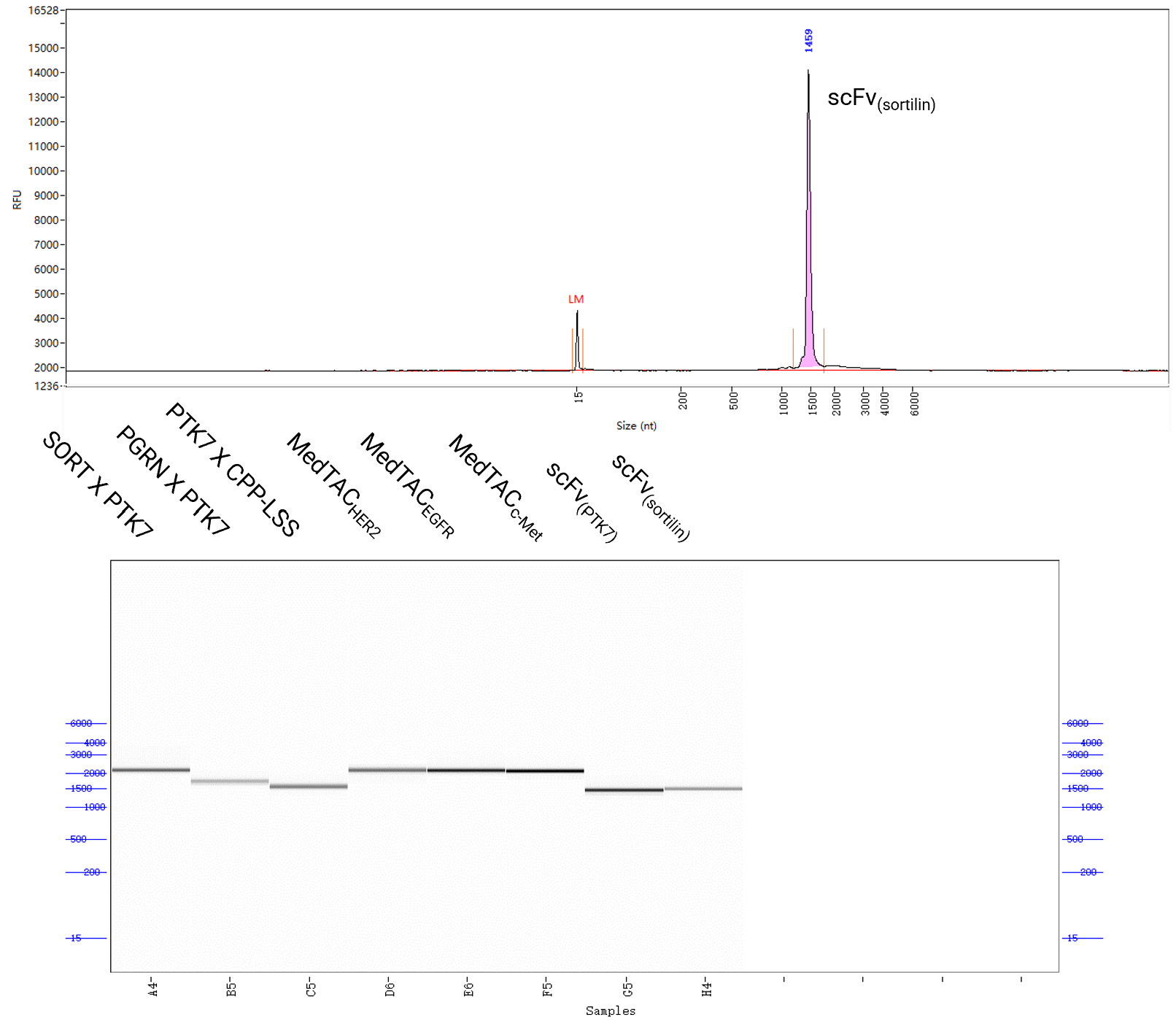


**Figure S2.** Characterization of template plasmids and IVT mRNA. (a) The integrity of circular and linearized plasmids was confirmed by gel electrophoresis. (b) The quality and purity of IVT mRNAs were assessed using agarose gel electrophoresis. (c) The integrity of IVT mRNAs was analyzed on an Agilent 5400 Bioanalyzer.

**
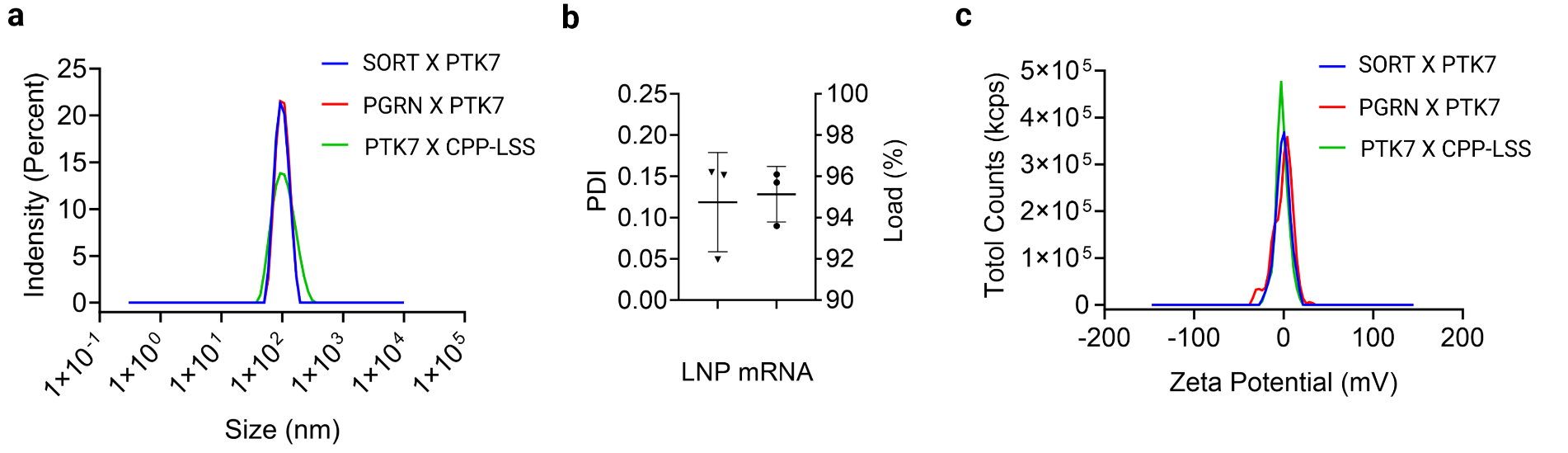
**

**Figure S3.** Characterization of LNP-encapsulated mRNA. (a) Particle size distribution of LNP-encapsulated mRNA. (b) Polydispersity index (PDI) and mRNA encapsulation efficiency, represented as mean ± standard deviation (n = 3 replicates). (c) Zeta potential measurement of LNP-encapsulated mRNA.

**
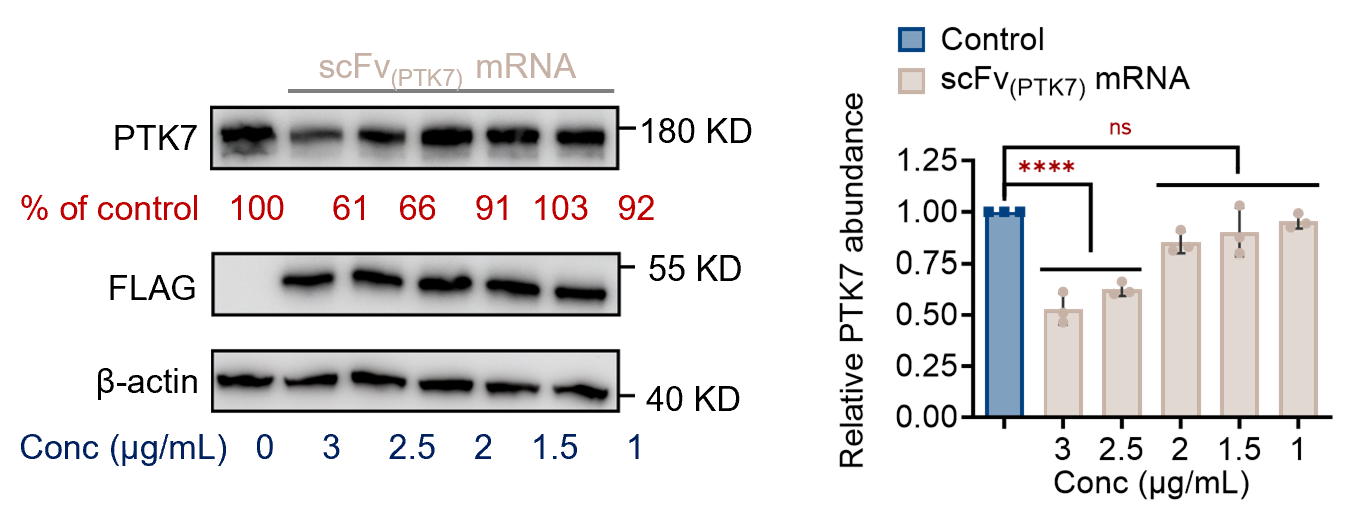
**

**Figure S4.** Evaluation of mRNA-encoding scFv(_PTK7_) for targeted PTK7 degradation. Western blot analysis of PTK7 levels in MDA-MB-468 cells treated with gradient concentrations of mRNA-encoding scFv(_PTK7_) for 48 hours, presented as the mean ± SD (n = 3). Statistical significance was determined by ordinary one-way ANOVA. n.s., not significant; ****p < 0.0001.


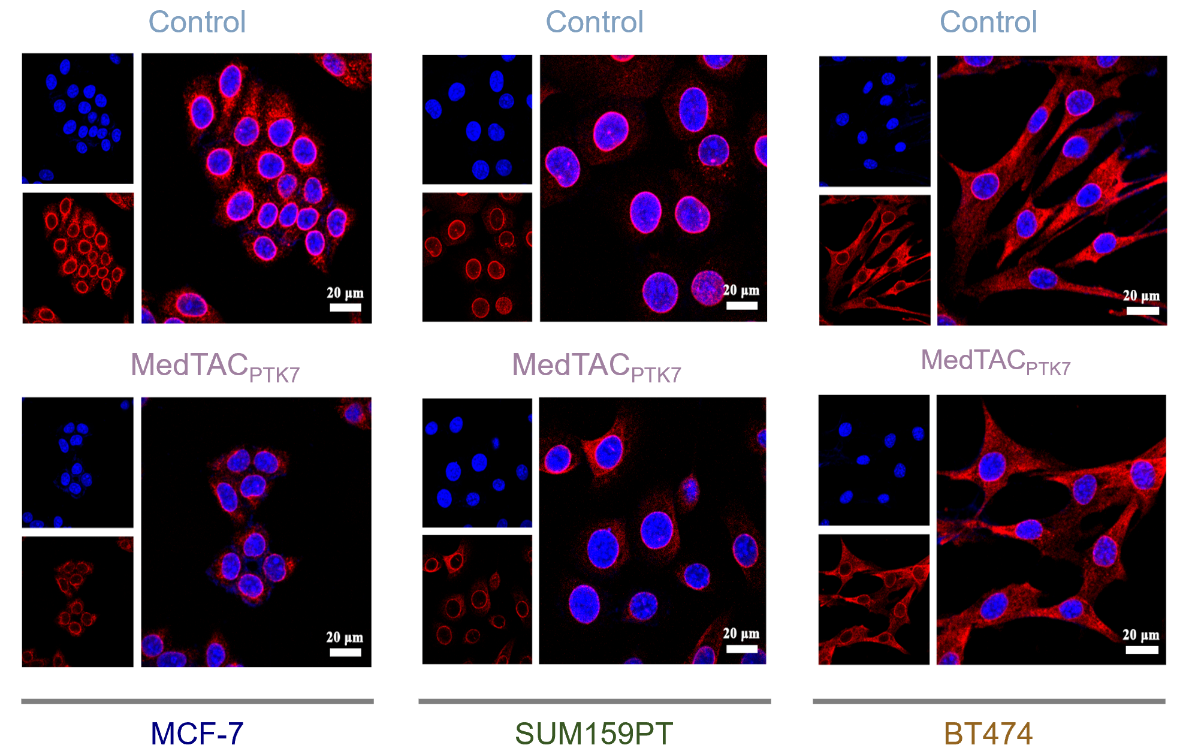


**Figure S5.** MedTAC_PTK7_-mediated degradation of PTK7 in a sortilin-dependent manner. Representative immunofluorescence images of MCF-7, SUM159PT and BT474 cells after 48 hours of treatment with 2 μg/mL MedTAC_PTK7_ or control (PTK7 in red; DAPI in blue). Scale bar: 20 μm.


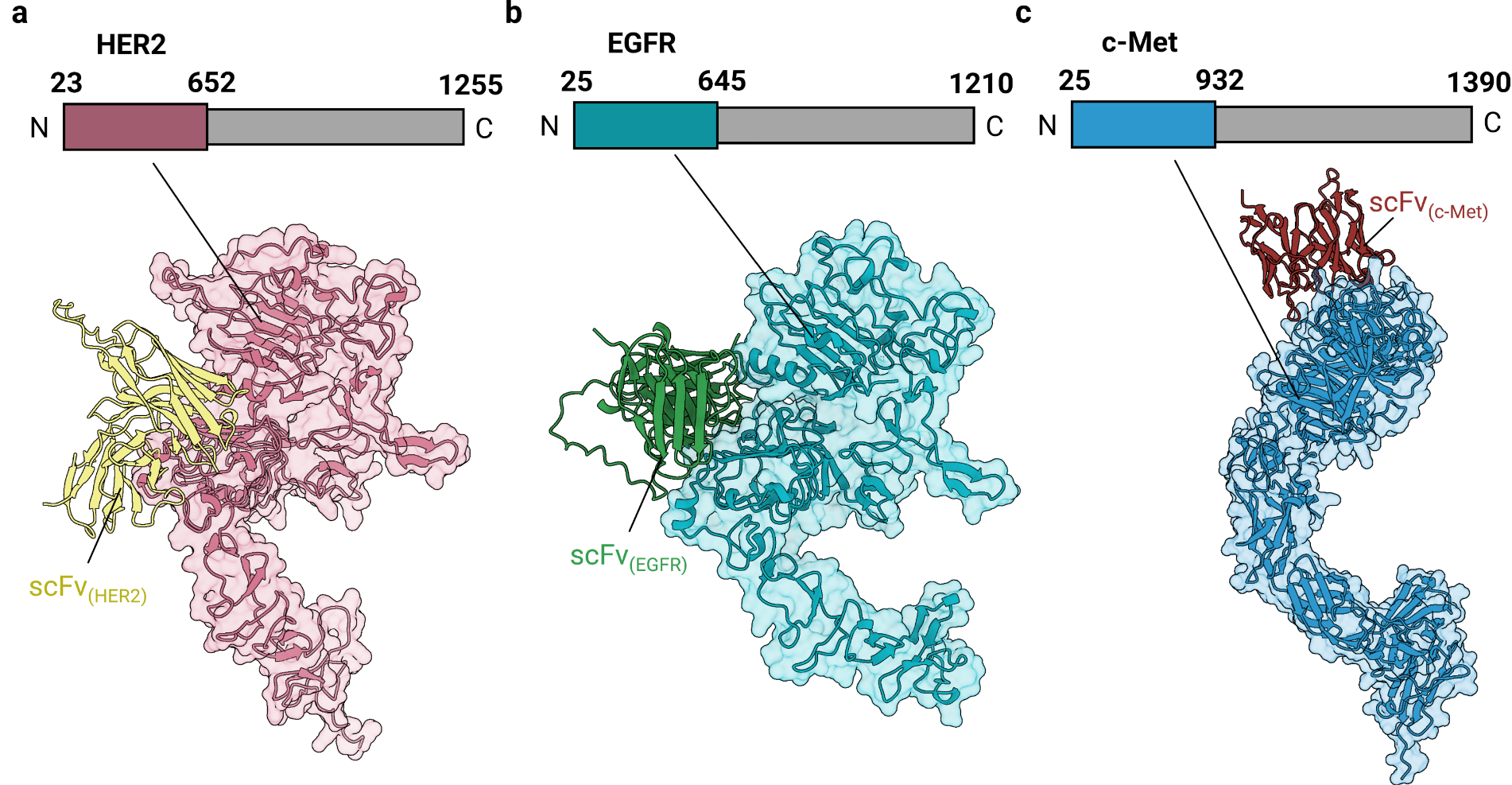


**Figure S6.** AlphaFold-Multimer predictions of the complex structures between designed scFvs and target proteins. (a) AlphaFold-Multimer predictions of the scFv_(HER2)_ derived from trastuzumab (Herceptin) with the HER2 extracellular domain, with HER2 in muted rose and scFv_(HER2)_ in pale yellow. (b) AlphaFold-Multimer predictions of the scFv_(EGFR)_ derived from cetuximab with the EGFR extracellular domain, with EGFR in cyan and scFv_(EGFR)_ in green. **(**c) AlphaFold-Multimer predictions of the scFv_(c-Met)_ derived from cetuximab with the c-Met extracellular domain, with c-Met in blue and scFv_(c-Met)_ in deep red.


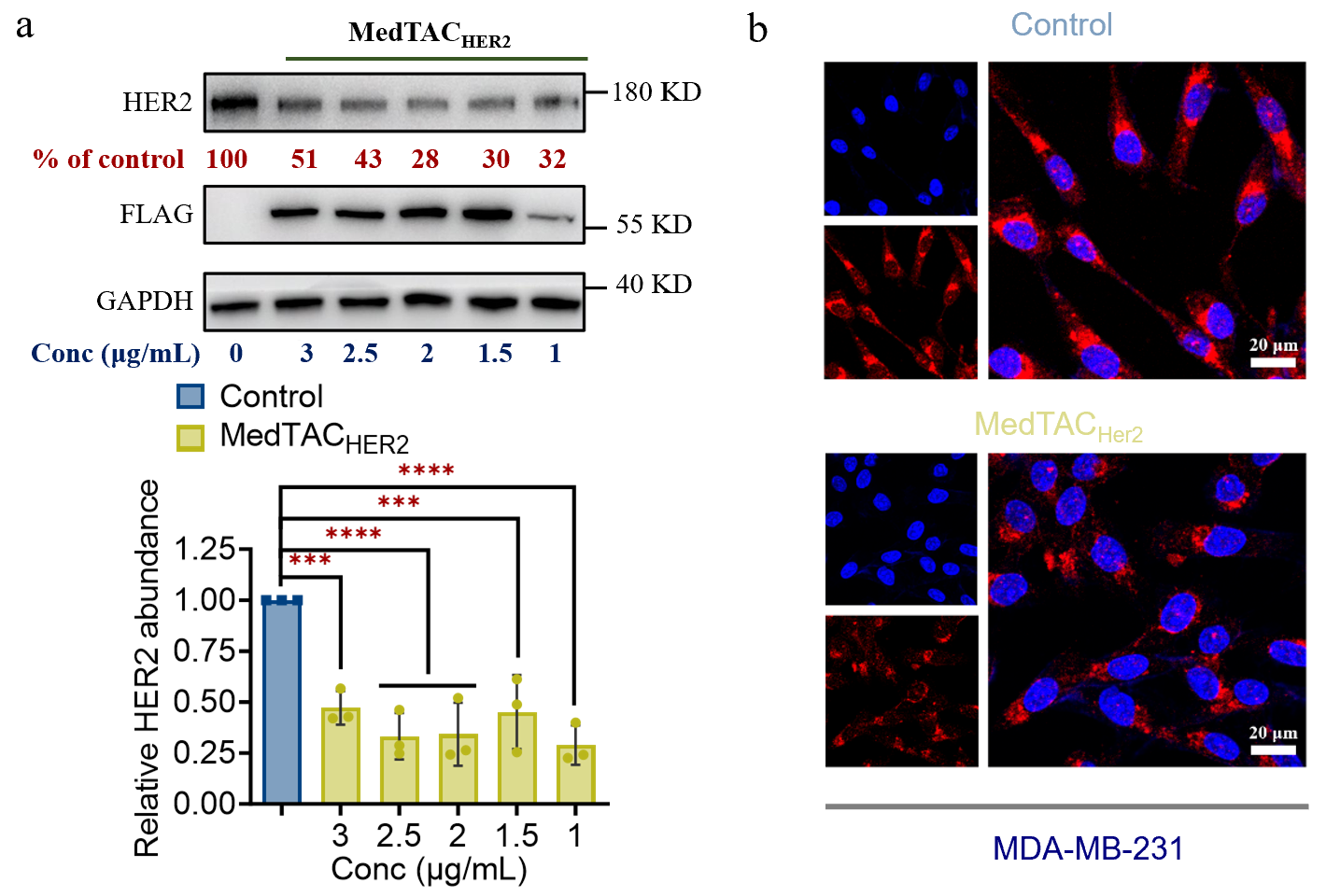


**Figure S7.** MedTAC_HER2_-mediated degradation of HER2 in MDA-MB-231 cells. (a**)** Western blot analysis of HER2 levels in MDA-MB-231 cells treated with gradient concentrations of MedTAC_HER2_. Quantitative data are presented as the mean ± SD (n = 3). All the p values were determined by ordinary one-way ANOVA. n.s., not significant; ***p < 0.001, ****p < 0.0001. (b) Representative immunofluorescence images of MDA-MB-231 cells after 48 hours of treatment with 2 μg/mL MedTAC_HER2_ or control (HER2 in red; DAPI in blue). Scale bar: 20 μm.

**
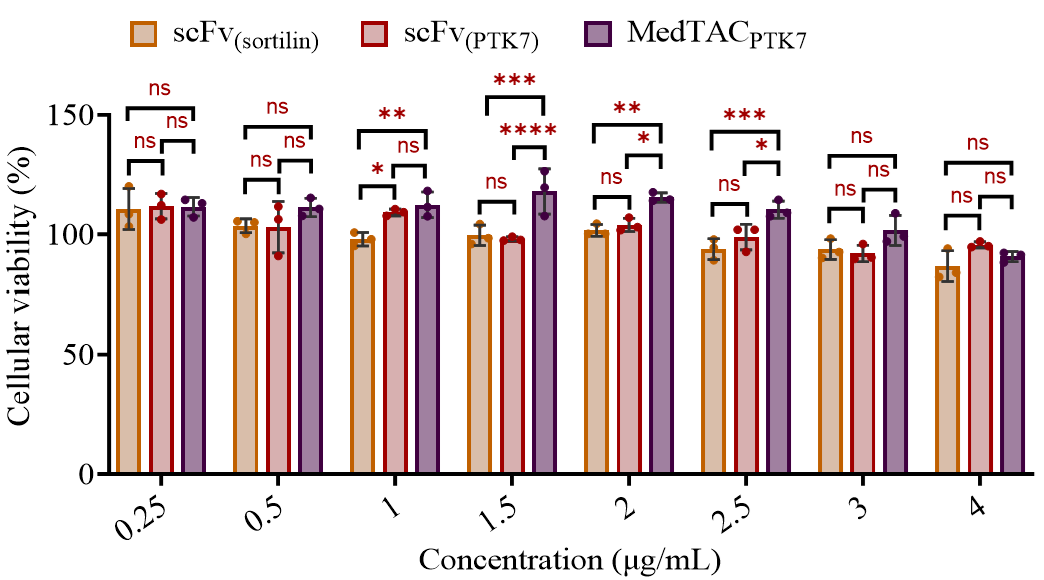
**

**Figure S8.** Evaluation of cell viability following treatment with MedTAC_PTK7_, mRNA-encoding scFv_(PTK7)_ and mRNA-encoding scFv_(sortilin)_. Data of Cell Counting Kit-8 (CCK8) assay are presented as mean ± SD, n = 3 per group. Statistical significance was determined by two-way ANOVA. n.s., not significant; *p < 0.05, **p < 0.01, ***p < 0.001, ****p < 0.0001.

**
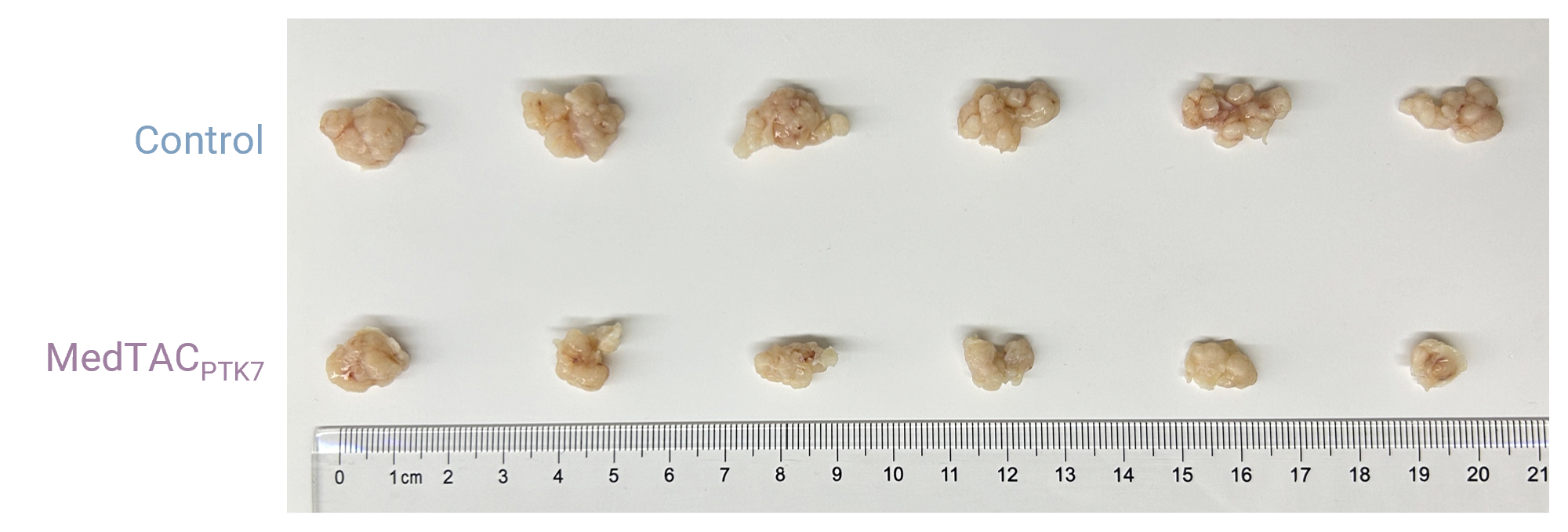
**

**Figure S9.** Analysis of tumor sizes in BALB/c nude mice (n=6) following three doses of DPBS or MedTAC_PTK7_ treatment.

**
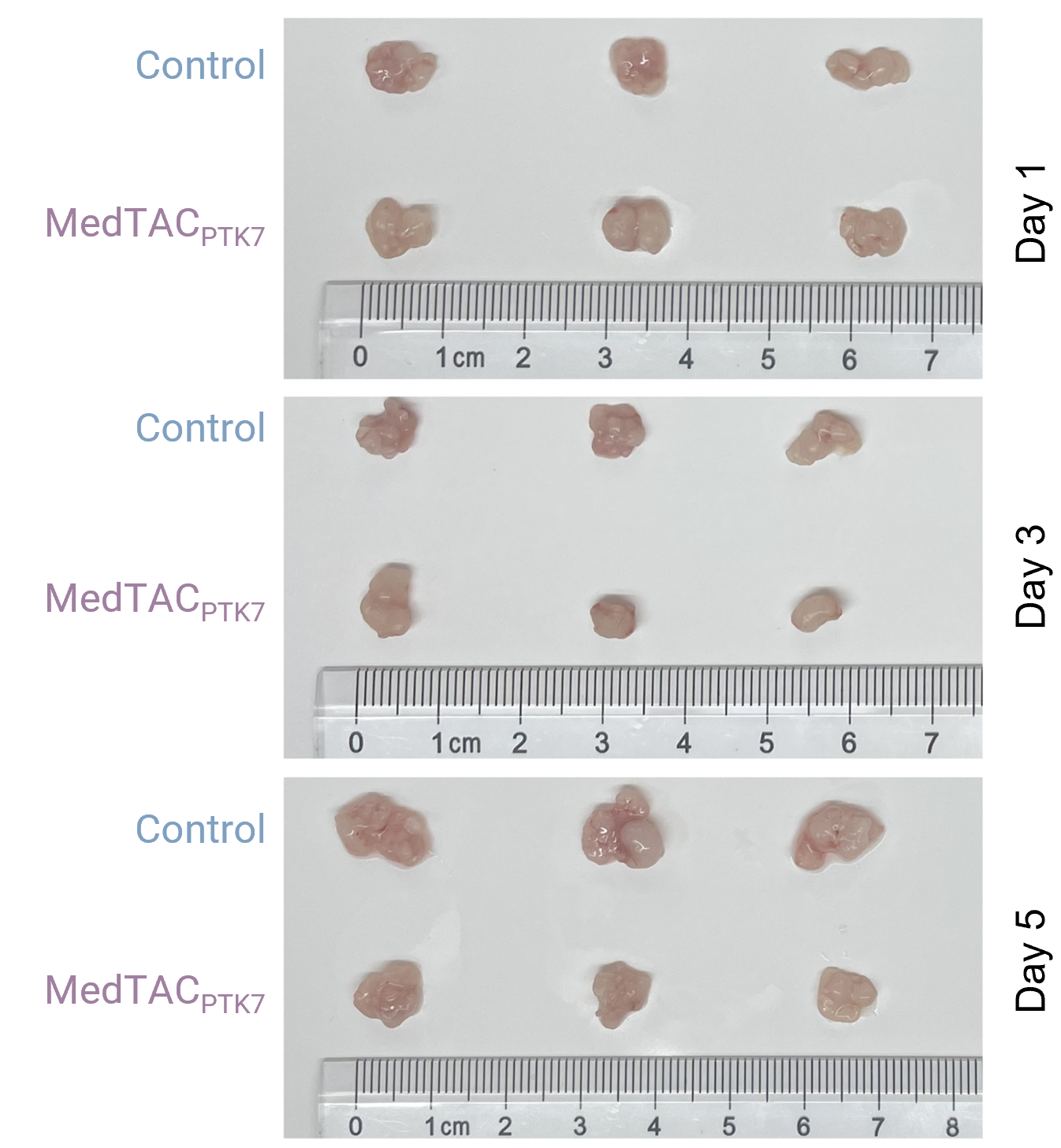
**

**Figure S10.** Comparison of tumor sizes on days 1, 3, and 5 following a single dose of DPBS or MedTAC_PTK7_ treatment.


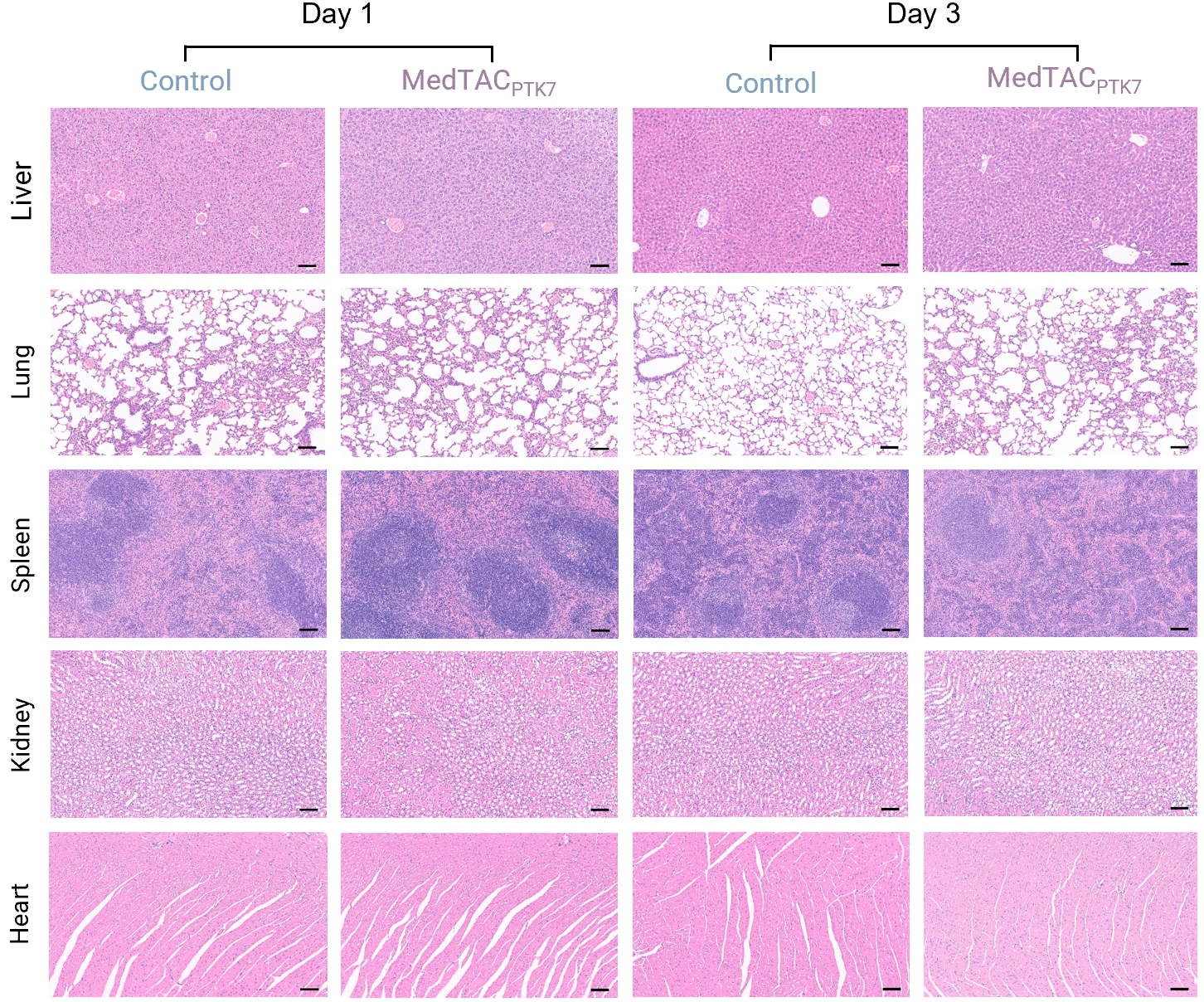


**Figure S11.** Hematoxylin-eosin staining (H&E) staining of main organs. H&E staining of main organs at day 1 and 3 after one-dose treatment of DPBS and MedTAC_PTK7_, respectively. Scale bars: 50 μm (20× magnification).

**Table S1.** Summary of LTRs and surface E3 ligase for extracellular and membrane-associated protein degradation technologies.

| **LTRs** | **Tissue distribution** | **Limitations** |
| --- | --- | --- |
| CI-MPR/  IGF2R ^[1]^ | Brain (medium)  Endocrine tissues (medium)  Respiratory tissues (high)  Gastrointestinal tract (medium)  Liver & Gallbladder (medium)  Pancreas (medium)  Kidney & Urinary bladder (medium)  Muscle tissues (medium)  Connective & Soft tissue (medium)  Skin (medium)  Bone marrow & Lymphoid tissues (medium) | 1. Widespread tissue distribution  2. Limited tumor specificity  3. Moderate expression in liver |
| ASGPR ^[2]^ | Liver & Gallbladder (high) | Limited to liver |
| E3 Ligase  (RNF43) ^[3]^ | Brain (high)  Endocrine tissues (high)  Respiratory tissues (high)  Proximal digestive tract (high)  Gastrointestinal tract (high)  Liver & Gallbladder (high)  Pancreas (high)  Kidney & Urinary bladder (high)  Muscle tissues (high)  Connective & Soft tissue (medium)  Skin (high)  Bone marrow & Lymphoid tissues (high) | 1. Widespread tissue distribution  2. Loss of function (mutation) in many cancer cells  3. poor tumor specificity  4. High expression in liver |
| Cytokine receptor ^[4]^ | Immune cells, primarily on activated T cells and some cancer cells  Overexpressed in many types of cancer | Potentially affects immune cells |
| Transferrin receptor  (TfR1) ^[5]^ | Brain (medium)  Endocrine tissues (medium)  Respiratory tissues (high)  Proximal digestive tract (medium)  Gastrointestinal tract (medium)  Kidney & Urinary bladder (medium)  Muscle tissues (medium)  Skin (medium)  Bone marrow & Lymphoid tissues (high)  Overexpressed in many types of cancer | Widespread tissue distribution |
| SCARA ^[6]^ | Brain (medium)  Endocrine tissues (high)  Respiratory tissues (medium)  Proximal digestive tract (medium)  Gastrointestinal tract (high)  Liver & Gallbladder (high)  Kidney & Urinary bladder (medium)  Muscle tissues (medium)  Bone marrow & Lymphoid tissues (high)  Overexpressed in some tumors | 1. Widespread tissue distribution  2. Typically found on macrophages  3. High expression in liver |
| Integrin ^[7]^ | Brain (medium)  Endocrine tissues (high)  Respiratory tissues (medium)  Proximal digestive tract (medium)  Gastrointestinal tract (medium)  Liver & Gallbladder (medium)  Pancreas (medium)  Kidney & Urinary bladder (high)  Muscle tissues (medium)  Bone marrow & Lymphoid tissues (high)  overexpressed in tumors, including αvβ3, αvβ5, and α5β1 | 1. Medium expression in liver  2. Widespread tissue distribution |

**Table S2.** Summary of predicted structural metrics for complexes.

| **Protein Complex** | **pLDDT** | **pTM** | **ipTM** |
| --- | --- | --- | --- |
| PGRN_493-593_/sortilin | 78.9 | 0.824 | 0.495 |
| NTS_24-170_/sortilin | 75.4 | 0.783 | 0.386 |
| NGF_122-241_/sortilin | 76.7 | 0.779 | 0.219 |
| BDNF_129-247_/sortilin | 78.8 | 0.782 | 0.219 |
| scFv_(sortilin)_/sortilin | 82.9 | 0.682 | 0.172 |
| scFv_(PTK7)_/PTK7 | 89.6 | 0.636 | 0.815 |
| scFv_(HER2)_/HER2 | 85.8 | 0.655 | 0.165 |
| scFv_(EGFR)_/EGFR | 84.6 | 0.547 | 0.457 |
| scFv_(c-Met)_/c-Met | 85.8 | 0.663 | 0.228 |

pLDDT: Predicted Local Distance Difference Test; pTM: Predicted Template Modeling score; ipTM: Interface Predicted Template Modeling score

**Table S3.** Codon-optimized DNA sequences of designed open reading frame.

| **Constructs** | **Codon-optimized DNA Sequence** |
| --- | --- |
| SORT X PTK7 | ATGGGCTGGTCCTGCATCATCTTGTTCTTGGTGGCTACTGCCACTGGAGTACACAGCCAAGTGCAGCTGCAGGAGAGCGGCCCTGGCCTGGTGAAGCCTAGCGAGACACTGAGCCTCACCTGTGCCGTTTCCGGCTACAGCATCAGCTCTGGATATTACTGGGGCTGGATCCGGCAGCCTCCAGGCAAGGGCCTGGAATGGATCGGCACAATCTACCACAGCGGCAGCACCTACTACAACCCCAGCCTGAAAAGCAGAGTGACCATCAGCGTGGACACAAGCAAGAACCAGTTCAGCCTGAAGCTGTCTTCTGTGACAGCCGCTGATACCGCCGTCTACTACTGCGCCAGACAGGGATCTATTAAGCAGGGCTACTACGGCATGGACGTGTGGGGACAGGGCACCACCGTGACCGTGTCCTCCGGAGGAGGCGGCTCTGGAGGTGGCGGCAGTGGTGGAGGCGGGTCTGGCGGTGGCGGATCTGGAGGTGGTGGGAGCGATATCGTGATGACCCAGTCCCCACTGAGCCTGCCTGTGACACCTGGCGAGCCTGCCAGCATCAGCTGTAGAAGCAGCCAGAGCCTGCTGAGATCCACCGGCTACAACTACCTGGACTGGTATCTCCAGAAACCCGGCCAGAGCCCCCAGCTGCTGATCTACCTGGGCAGCAACCGGGCCTCTGGCGTGCCTGATAGATTCAGCGGATCTGGAAGCGGCACCGACTTCACCCTGAAGATCTCTAGAGCTGAAGCCGAGGACGTGGGCGTCTACTACTGCATGCAGCAACAGGAGGCCCCTCTGACCTTTGGCGGCGGAACAAAGGTGGAAATCAAGCGGAGTGGCGGCGGAGGATCCCAAGTGCAGCTGGTGCAGAGCGGCCCTGAGGTGAAGAAGCCCGGCGCTTCTGTGAAAGTGTCCTGCAAGGCCAGCGGCTACACCTTTACCGATTACGCCGTGCACTGGGTGCGGCAGGCCCCTGGAAAGCGCCTGGAATGGATCGGCGTGATCAGCACCTACAACGACTATACATACAACAACCAGGACTTCAAGGGCAGAGTGACCATGACAAGAGATACCAGCGCCTCTACCGCCTACATGGAACTGAGCAGACTGAGAAGCGAGGACACCGCTGTCTACTACTGTGCCCGGGGCAATAGCTACTTCTACGCCCTGGACTACTGGGGACAGGGCACATCTGTTACAGTGTCCAGCGGAGGCGGAGGATCTGGTGGTGGCGGATCTGGCGGCGGTGGAAGTGGCGGAGGTGGTAGCGAGATCGTGCTGACACAGAGCCCTGCCACCCTCTCCCTGTCCCCAGGCGAGCGGGCCACACTGTCTTGTAGAGCCAGCGAGAGCGTGGACAGCTACGGCAAGTCCTTCATGCACTGGTATCAGCAGAAACCTGGCCAGGCCCCTAGACTGCTGATCTACAGAGCTAGCAACCTGGAAAGCGGCATCCCTGCTAGATTCAGCGGCAGCGGATCTGGCACAGACTTCACCCTGACCATCAGCAGCCTGGAACCCGAGGATTTTGCCGTGTACTACTGCCAGCAATCTAACGAGGACCCCTGGACCTTCGGCGGCGGAACCAAGCTGGAAATCAAGCGGGGCGGCTCTGACTACAAGGACGACGATGATAAG |
| PGRN X PTK7 | ATGGGCTGGTCCTGCATCATCTTGTTCTTGGTGGCTACTGCCACTGGAGTACACAGCCGCAGCTGCGAGAAAGAGGTGGTGTCTGCCCAGCCTGCTACATTTCTGGCCAGAAGCCCCCACGTGGGAGTCAAGGACGTGGAATGCGGCGAGGGCCATTTCTGCCACGACAACCAGACCTGTTGTAGAGATAACAGACAAGGCTGGGCCTGCTGCCCTTACAGACAGGGCGTGTGCTGTGCCGATAGACGGCACTGCTGTCCTGCCGGCTTCCGGTGCGCCGCTAGAGGCACCAAGTGCCTGAGGCGGGAAGCCCCAAGATGGGACGCCCCTCTGCGGGACCCCGCCCTGAGACAGCTGCTGAGTGGCGGCGGAGGATCCCAAGTGCAGCTGGTGCAGAGCGGCCCTGAGGTGAAGAAGCCCGGCGCTTCTGTGAAAGTGTCCTGCAAGGCCAGCGGCTACACCTTTACCGATTACGCCGTGCACTGGGTGCGGCAGGCCCCTGGAAAGCGCCTGGAATGGATCGGCGTGATCAGCACCTACAACGACTATACATACAACAACCAGGACTTCAAGGGCAGAGTGACCATGACAAGAGATACCAGCGCCTCTACCGCCTACATGGAACTGAGCAGACTGAGAAGCGAGGACACCGCTGTCTACTACTGTGCCCGGGGCAATAGCTACTTCTACGCCCTGGACTACTGGGGACAGGGCACATCTGTTACAGTGTCCAGCGGAGGCGGAGGATCTGGTGGTGGCGGATCTGGCGGCGGTGGAAGTGGCGGAGGTGGTAGCGAGATCGTGCTGACACAGAGCCCTGCCACCCTCTCCCTGTCCCCAGGCGAGCGGGCCACACTGTCTTGTAGAGCCAGCGAGAGCGTGGACAGCTACGGCAAGTCCTTCATGCACTGGTATCAGCAGAAACCTGGCCAGGCCCCTAGACTGCTGATCTACAGAGCTAGCAACCTGGAAAGCGGCATCCCTGCTAGATTCAGCGGCAGCGGATCTGGCACAGACTTCACCCTGACCATCAGCAGCCTGGAACCCGAGGATTTTGCCGTGTACTACTGCCAGCAATCTAACGAGGACCCCTGGACCTTCGGCGGCGGAACCAAGCTGGAAATCAAGCGGGGCGGCTCTGACTACAAGGACGACGATGATAAG |
| PTK7 X CPP-LSS | ATGGGCTGGTCCTGCATCATCCTGTTCCTCGTCGCCACAGCCACCGGCGTGCACAGCGACTACAAGGACGACGATGATAAGTCTGGAGGCGGAGGCAGCCAGGTTCAGCTGGTGCAGTCTGGACCTGAGGTGAAAAAGCCTGGCGCCAGCGTGAAGGTGTCTTGCAAGGCCAGCGGCTATACATTCACCGACTACGCAGTCCACTGGGTGCGGCAGGCTCCAGGCAAAAGACTGGAATGGATCGGCGTGATCAGCACCTACAACGACTACACCTACAATAACCAGGACTTTAAGGGCAGGGTGACCATGACCAGAGATACAAGCGCCAGCACCGCTTACATGGAACTGAGCAGACTGAGATCCGAGGACACCGCCGTGTACTACTGCGCCCGGGGCAACAGCTACTTCTACGCCCTGGACTACTGGGGACAAGGCACAAGCGTGACCGTGTCCAGCGGGGGCGGCGGAAGCGGCGGCGGAGGATCTGGCGGCGGCGGAAGCGGCGGCGGCGGCTCCGAGATCGTGCTGACCCAGAGCCCTGCCACCCTGAGCCTGTCCCCAGGCGAGCGGGCCACACTGTCTTGTAGAGCTTCTGAGAGCGTGGACAGCTACGGCAAGAGCTTCATGCACTGGTACCAGCAGAAGCCCGGACAGGCCCCTAGACTGCTGATCTACAGAGCCAGCAATCTGGAAAGCGGCATCCCCGCTAGATTCTCTGGCAGCGGTAGCGGAACAGATTTTACCCTGACAATCTCAAGCCTGGAACCTGAGGACTTCGCCGTGTACTACTGCCAGCAAAGCAACGAGGATCCTTGGACTTTCGGCGGCGGCACCAAGCTGGAAATTAAGCGGAGCGGCGGCGGCGGCTCCGGCGGCGGCAGACGGAGAAGACGGCGGAGACGCAGAAACCCCGGCTAT |
| MedTAC_Her2_ | ATGGGCTGGTCTTGCATCATCCTGTTCCTGGTGGCCACAGCCACCGGCGTGCACAGCCAAGTGCAGCTGCAGGAGAGCGGCCCTGGCCTGGTGAAGCCTAGCGAGACACTGAGCCTCACCTGTGCCGTTTCCGGCTACAGCATCAGCTCTGGATATTACTGGGGCTGGATCCGGCAGCCTCCAGGCAAGGGCCTGGAATGGATCGGCACAATCTACCACAGCGGCAGCACCTACTACAACCCCAGCCTGAAAAGCAGAGTGACCATCAGCGTGGACACAAGCAAGAACCAGTTCAGCCTGAAGCTGTCTTCTGTGACAGCCGCTGATACCGCCGTCTACTACTGCGCCAGACAGGGATCTATTAAGCAGGGCTACTACGGCATGGACGTGTGGGGACAGGGCACCACCGTGACCGTGTCCTCCGGAGGCGGAGGCAGCGGCGGCGGAGGCAGCGGCGGCGGCGGTTCCGGCGGCGGAGGCAGCGGCGGCGGCGGCTCTGATATCGTGATGACCCAGTCCCCACTGAGCCTGCCTGTGACACCTGGCGAGCCTGCCAGCATCAGCTGTAGAAGCAGCCAGAGCCTGCTGAGATCCACCGGCTACAACTACCTGGACTGGTATCTCCAGAAACCCGGCCAGAGCCCCCAGCTGCTGATCTACCTGGGCAGCAACCGGGCCTCTGGCGTGCCTGATAGATTCAGCGGATCTGGAAGCGGCACCGACTTCACCCTGAAGATCTCTAGAGCTGAAGCCGAGGACGTGGGCGTCTACTACTGCATGCAGCAACAGGAGGCCCCTCTGACCTTTGGCGGCGGAACAAAGGTGGAAATCAAGCGGTCTGGCGGCGGAGGCAGCGAGGTGCAGCTGGTGGAAAGCGGAGGAGGCCTGGTGCAGCCTGGAGGCAGCCTCAGACTGTCCTGCGCCGCTTCCGGCTTTAATATCAAGGACACCTACATCCACTGGGTGCGGCAGGCCCCAGGCAAGGGCCTGGAATGGGTGGCCAGAATCTACCCCACAAACGGCTACACAAGATACGCCGACAGCGTCAAGGGCAGATTCACCATCTCCGCTGACACCAGCAAGAACACCGCCTACCTGCAGATGAACAGCCTGCGGGCCGAGGACACAGCTGTGTACTATTGTAGCAGATGGGGCGGCGATGGATTTTACGCCATGGACTACTGGGGCCAGGGCACCCTGGTCACCGTGTCCAGCGGCGGCGGCGGAAGCGGCGGCGGCGGCTCTGGCGGAGGCGGATCTGGCGGCGGCGGCAGTGATATCCAGATGACACAGAGCCCCAGCTCTCTGAGCGCTTCTGTGGGAGATAGAGTGACAATCACCTGCAGAGCCTCTCAGGACGTGAACACAGCCGTGGCCTGGTACCAGCAGAAACCTGGCAAAGCCCCTAAGCTGCTGATCTATAGCGCCAGCTTCCTGTACAGCGGTGTTCCTAGCAGGTTCAGCGGCAGCCGGAGCGGAACAGACTTCACACTGACCATTTCTAGCCTGCAGCCCGAGGATTTCGCCACCTACTACTGCCAGCAACACTACACCACCCCTCCTACCTTCGGCCAAGGCACCAAGGTGGAGATCAAGCGGGGCGGCTCTGACTACAAGGACGACGATGATAAG |
| MedTAC_EGFR_ | ATGGGCTGGTCTTGCATCATCCTGTTCCTGGTGGCCACAGCCACCGGCGTGCACAGCCAAGTGCAGCTGCAGGAGAGCGGCCCTGGCCTGGTGAAGCCTAGCGAGACACTGAGCCTCACCTGTGCCGTTTCCGGCTACAGCATCAGCTCTGGATATTACTGGGGCTGGATCCGGCAGCCTCCAGGCAAGGGCCTGGAATGGATCGGCACAATCTACCACAGCGGCAGCACCTACTACAACCCCAGCCTGAAAAGCAGAGTGACCATCAGCGTGGACACAAGCAAGAACCAGTTCAGCCTGAAGCTGTCTTCTGTGACAGCCGCTGATACCGCCGTCTACTACTGCGCCAGACAGGGATCTATTAAGCAGGGCTACTACGGCATGGACGTGTGGGGACAGGGCACCACCGTGACCGTGTCCTCCGGAGGCGGAGGCAGCGGCGGCGGAGGCAGCGGCGGCGGCGGTTCCGGCGGCGGAGGCAGCGGCGGCGGCGGCTCTGATATCGTGATGACCCAGTCCCCACTGAGCCTGCCTGTGACACCTGGCGAGCCTGCCAGCATCAGCTGTAGAAGCAGCCAGAGCCTGCTGAGATCCACCGGCTACAACTACCTGGACTGGTATCTCCAGAAACCCGGCCAGAGCCCCCAGCTGCTGATCTACCTGGGCAGCAACCGGGCCTCTGGCGTGCCTGATAGATTCAGCGGATCTGGAAGCGGCACCGACTTCACCCTGAAGATCTCTAGAGCTGAAGCCGAGGACGTGGGCGTCTACTACTGCATGCAGCAACAGGAGGCCCCTCTGACCTTTGGCGGCGGAACAAAGGTGGAAATCAAGCGGTCTGGCGGCGGAGGCAGCCAGGTTCAACTGAAACAGAGCGGACCTGGCCTGGTCCAGCCTTCTCAGAGCCTGAGCATCTGCACCGTGAGCGGCTTCAGCCTGACAAATTACGGCGTGCACTGGGTGCGGCAGTCTCCTGGCAAAGGCCTCGAGTGGCTGGGCGTGATCTGGAGCGGCGGCAATACCGATTATAACACCCCTTTTACAAGCCGGCTGAGCATTAACAAGGACAACAGCAAGAGCCAGGTGTTCTTCAAGATGAACTCCCTGCAGAGCAACGACACCGCCATCTACTACTGCGCCAGAGCCCTGACCTACTACGACTACGAGTTCGCCTACTGGGGCCAGGGCACACTGGTGACCGTGAGCGCCGGCGGCGGAGGTTCTGGCGGCGGCGGATCTGGCGGAGGAGGGTCCGGCGGCGGCGGCAGCGACATCCTGCTGACACAGTCTCCCGTGATCCTGAGCGTGTCCCCAGGCGAGAGAGTGTCCTTTTCATGCAGAGCTAGCCAGAGCATCGGCACCAATATCCACTGGTACCAGCAAAGAACCAACGGCAGCCCTAGACTGCTGATCAAGTACGCCAGCGAGAGCATCTCCGGCATCCCCAGCAGGTTCAGCGGATCCGGAAGCGGCACAGACTTCACACTGTCTATCAACAGCGTGGAATCTGAAGATATCGCCGATTACTATTGTCAGCAGAACAACAACTGGCCCACCACCTTCGGCGCTGGCACCAAGCTGGAACTGAAGCGGGGCGGCTCTGACTACAAGGACGACGATGATAAG |
| MedTAC_c-Met_ | ATGGGCTGGTCTTGCATCATCCTGTTCCTGGTGGCCACAGCCACCGGCGTGCACAGCCAAGTGCAGCTGGTCCAGAGCGGCGCCGAGGTGAAAAAGCCTGGAGCTTCTGTGAAGATGAGCTGCAAGGCCTCTGGCTACACCTTCACCTCTTATTGGATGCACTGGGTGCGGCAGGCCCCTGGCCAGGGACTGGAATGGATCGGCGCTATCTACCCAGGCAACAGCGAGACAGGCTACGCCCAGAAGTTCCAGGGCAGAGCCACCCTGACCGCCGACACCTCCACCAGCACCGCCTACATGGAACTGAGCAGCCTGAGAAGCGAGGATACAGCCGTGTACTACTGTACAAGAGAGAACTGGGACCCCGGCTTCGCCTTTTGGGGCCAGGGAACACTGATCACCGTGTCCAGCGGCGGAGGTGGCTCTGGAGGCGGTGGATCTGGTGGTGGCGGATCAGGTGGAGGCGGGAGCGGAGGCGGCGGTAGTGATATCCAGATGACCCAGAGCCCCAGCAGCCTGAGCGCCTCTGTGGGCGACAGAGTGACCATCACCTGTAGCGCTTCTTCCTCCGTCTACTATATGTACTGGTTTCAGCAGAAACCTGGCAAGGCCCCTAAGCTGTGGATCTACAGCACCAGCAACCTGGCCAGCGGCGTGCCATCTAGATTCAGCGGCAGCGGATCTGGAACAGACTACACACTGACCATCAGCTCCATGCAGCCTGAGGACTTCGCCACATACTACTGCCAGCAAAGACGGAACTACCCCTACACCTTCGGCCAGGGCACCAAGCTGGAAATCAAGTCCGGTGGAGGAGGCTCTCAAGTGCAGCTGGTCCAGAGCGGTGCTGAGGTGAAAAAGCCCGGCGCCTCCGTGAAGGTGTCCTGCAAGGCCAGCGGCTACATCTTTACCGCCTATACAATGCACTGGGTGCGGCAGGCTCCTGGACAGGGCCTGGAATGGATGGGCTGGATCAAGCCCAACAACGGCCTGGCCAACTACGCCCAGAAGTTCCAGGGCAGGGTGACAATGACTAGAGATACCAGCATCAGCACCGCTTATATGGAACTGAGCCGCCTGAGAAGCGACGACACCGCCGTGTACTACTGTGCCAGATCTGAGATCACCACCGAGTTCGACTACTGGGGCCAGGGCACCCTGGTTACAGTGTCTTCTGGCGGAGGTGGCTCTGGAGGCGGTGGATCTGGTGGTGGCGGATCAGGTGGAGGCGGGAGCGACATCGTGATGACCCAGTCCCCTGATAGCCTGGCCGTGTCCCTCGGAGAGCGGGCTACAATTAACTGCAAGAGCAGCGAGTCTGTGGACAGCTACGCCAATTCTTTTCTGCACTGGTACCAGCAAAAACCCGGCCAGCCTCCTAAGCTGCTGATCTACCGGGCCAGCACAAGAGAGAGCGGCGTGCCAGATAGATTCAGCGGCAGCGGAAGCGGCACCGACTTCACCCTGACAATCAGCAGCCTGCAGGCCGAGGACGTGGCCGTCTACTACTGCCAGCAGAGCAAGGAAGATCCTCTGACCTTCGGCGGCGGAACCAAGGTGGAAATCAAAAGAGGCGGCTCTGACTACAAGGACGACGATGATAAG |
| scFv_(PTK7)_ | ATGGGCTGGTCCTGCATCATCTTGTTCTTGGTGGCTACTGCCACTGGAGTACACAGCCAAGTGCAGCTGGTGCAGAGCGGCCCTGAGGTGAAGAAGCCCGGCGCTTCTGTGAAAGTGTCCTGCAAGGCCAGCGGCTACACCTTTACCGATTACGCCGTGCACTGGGTGCGGCAGGCCCCTGGAAAGCGCCTGGAATGGATCGGCGTGATCAGCACCTACAACGACTATACATACAACAACCAGGACTTCAAGGGCAGAGTGACCATGACAAGAGATACCAGCGCCTCTACCGCCTACATGGAACTGAGCAGACTGAGAAGCGAGGACACCGCTGTCTACTACTGTGCCCGGGGCAATAGCTACTTCTACGCCCTGGACTACTGGGGACAGGGCACATCTGTTACAGTGTCCAGCGGAGGCGGAGGATCTGGTGGTGGCGGATCTGGCGGCGGTGGAAGTGGCGGAGGTGGTAGCGAGATCGTGCTGACACAGAGCCCTGCCACCCTCTCCCTGTCCCCAGGCGAGCGGGCCACACTGTCTTGTAGAGCCAGCGAGAGCGTGGACAGCTACGGCAAGTCCTTCATGCACTGGTATCAGCAGAAACCTGGCCAGGCCCCTAGACTGCTGATCTACAGAGCTAGCAACCTGGAAAGCGGCATCCCTGCTAGATTCAGCGGCAGCGGATCTGGCACAGACTTCACCCTGACCATCAGCAGCCTGGAACCCGAGGATTTTGCCGTGTACTACTGCCAGCAATCTAACGAGGACCCCTGGACCTTCGGCGGCGGAACCAAGCTGGAAATCAAGCGGGGCGGCTCTGACTACAAGGACGACGATGATAAG |
| scFv_(sortilin)_ | ATGGGCTGGTCCTGCATCATCTTGTTCTTGGTGGCTACTGCCACTGGAGTACACAGCCAAGTGCAGCTGCAGGAGAGCGGCCCTGGCCTGGTGAAGCCTAGCGAGACACTGAGCCTCACCTGTGCCGTTTCCGGCTACAGCATCAGCTCTGGATATTACTGGGGCTGGATCCGGCAGCCTCCAGGCAAGGGCCTGGAATGGATCGGCACAATCTACCACAGCGGCAGCACCTACTACAACCCCAGCCTGAAAAGCAGAGTGACCATCAGCGTGGACACAAGCAAGAACCAGTTCAGCCTGAAGCTGTCTTCTGTGACAGCCGCTGATACCGCCGTCTACTACTGCGCCAGACAGGGATCTATTAAGCAGGGCTACTACGGCATGGACGTGTGGGGACAGGGCACCACCGTGACCGTGTCCTCCGGAGGAGGCGGCTCTGGAGGTGGCGGCAGTGGTGGAGGCGGGTCTGGCGGTGGCGGATCTGGAGGTGGTGGGAGCGATATCGTGATGACCCAGTCCCCACTGAGCCTGCCTGTGACACCTGGCGAGCCTGCCAGCATCAGCTGTAGAAGCAGCCAGAGCCTGCTGAGATCCACCGGCTACAACTACCTGGACTGGTATCTCCAGAAACCCGGCCAGAGCCCCCAGCTGCTGATCTACCTGGGCAGCAACCGGGCCTCTGGCGTGCCTGATAGATTCAGCGGATCTGGAAGCGGCACCGACTTCACCCTGAAGATCTCTAGAGCTGAAGCCGAGGACGTGGGCGTCTACTACTGCATGCAGCAACAGGAGGCCCCTCTGACCTTTGGCGGCGGAACAAAGGTGGAAATCAAGCGGGGCGGCTCTGACTACAAGGACGACGATGATAAG |

Appended Western Blotting Figures

1.
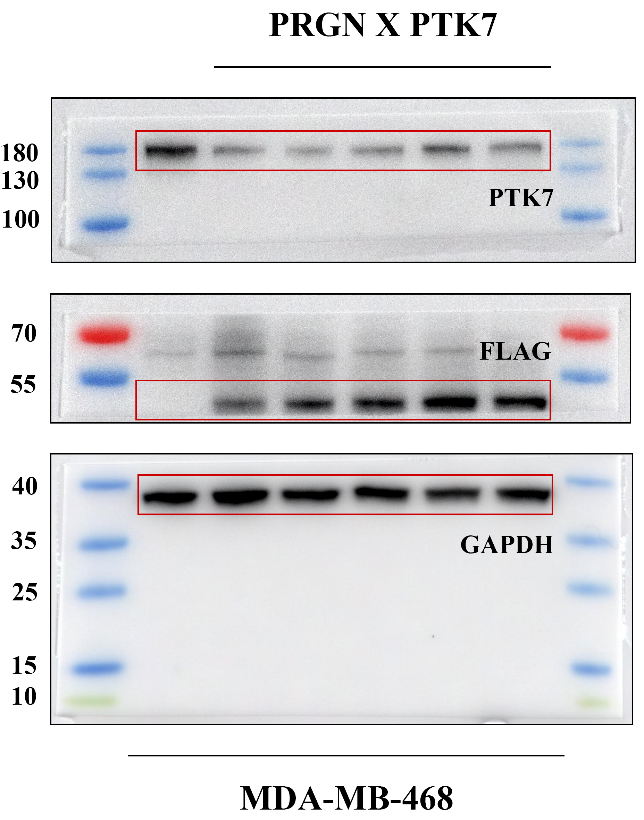
The original western blotting images for Figure 2e.


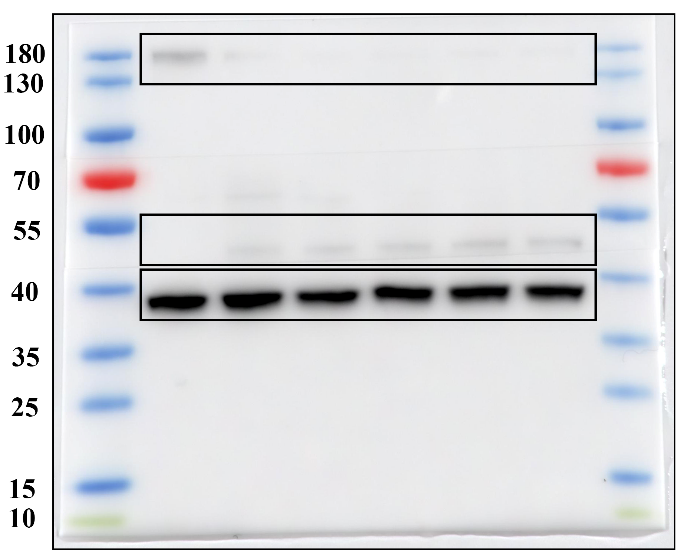


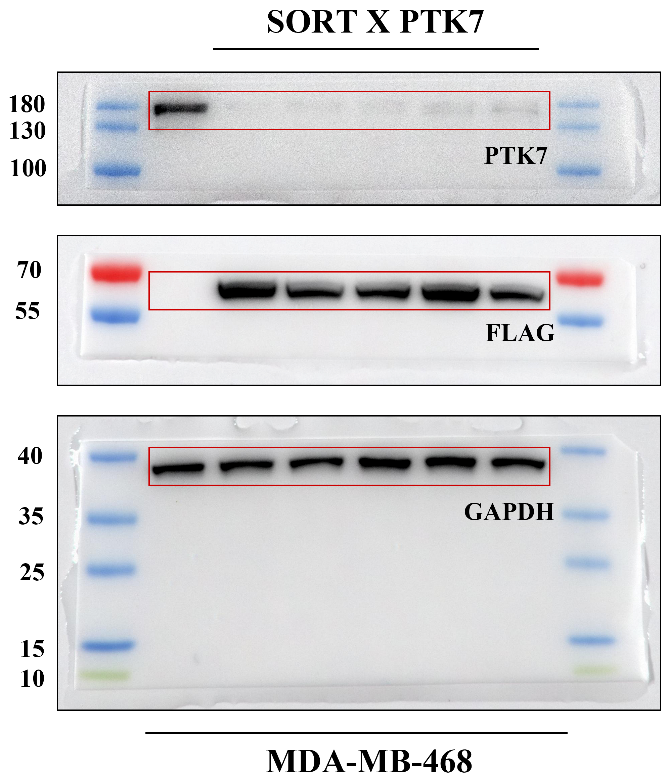


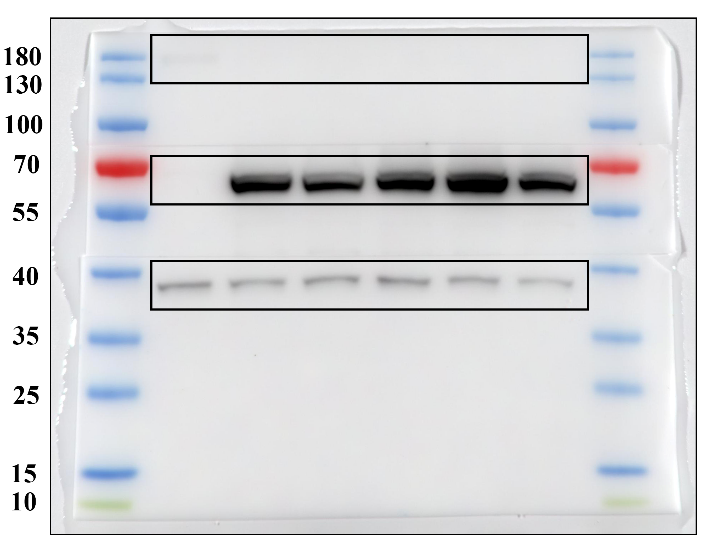


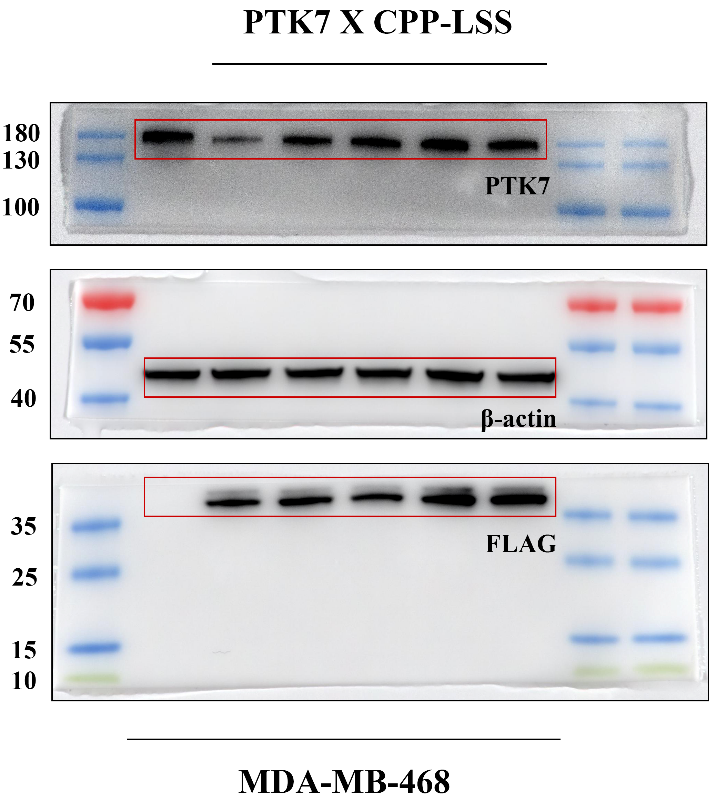


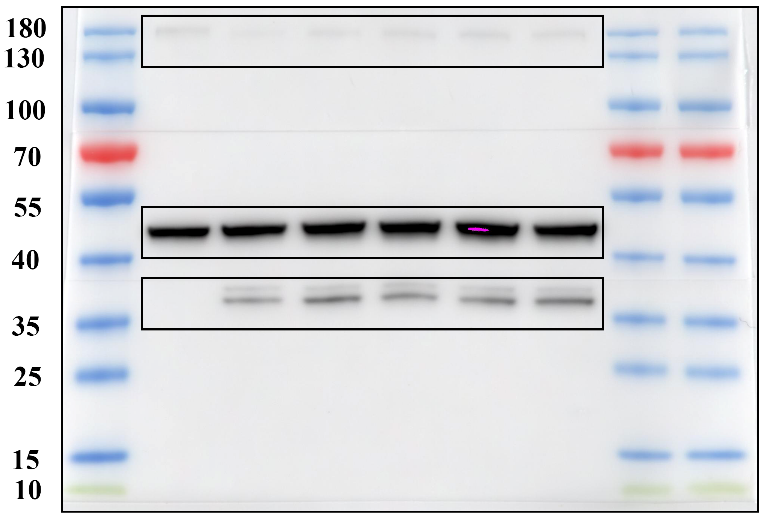


1. The original western blotting images for Figure 3a.


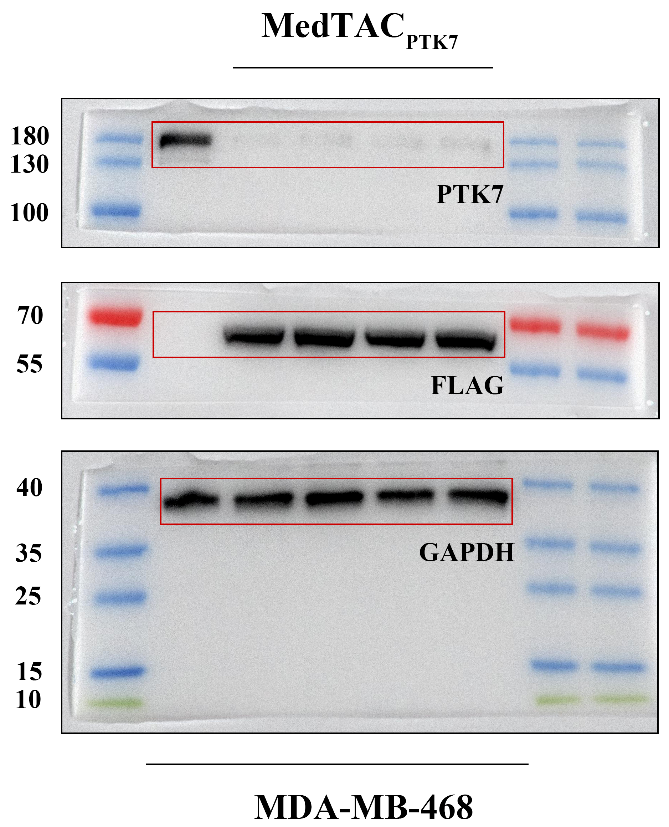


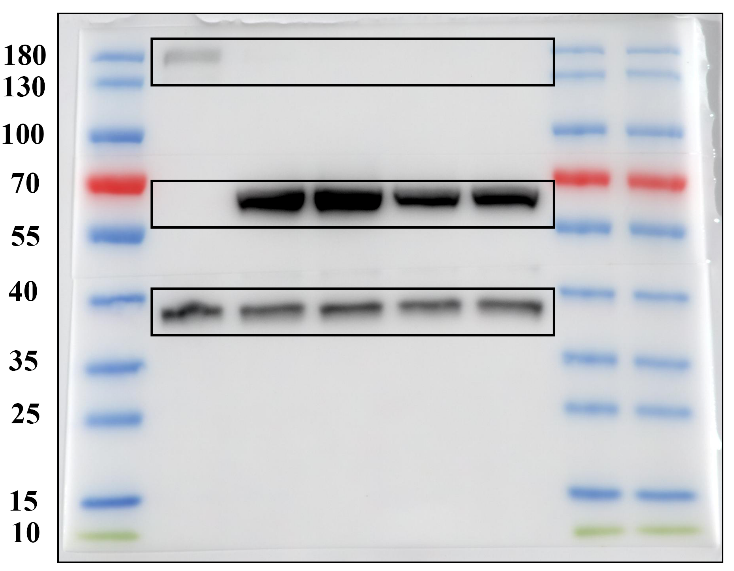


1.
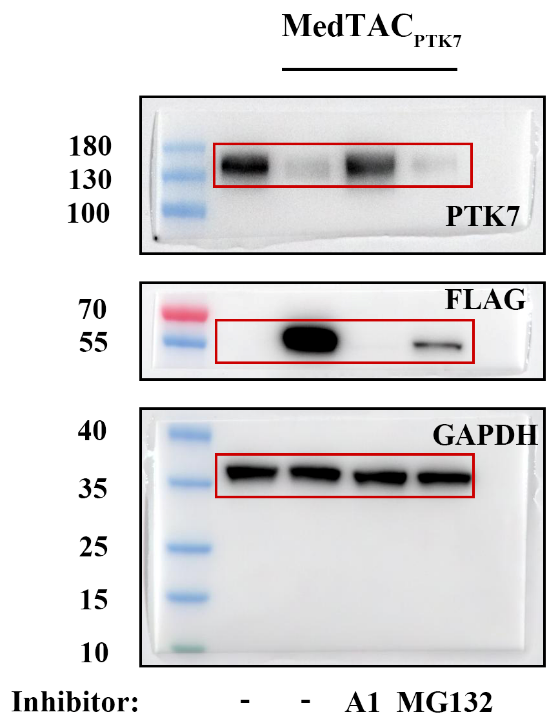
The original western blotting images for Figure 3b.


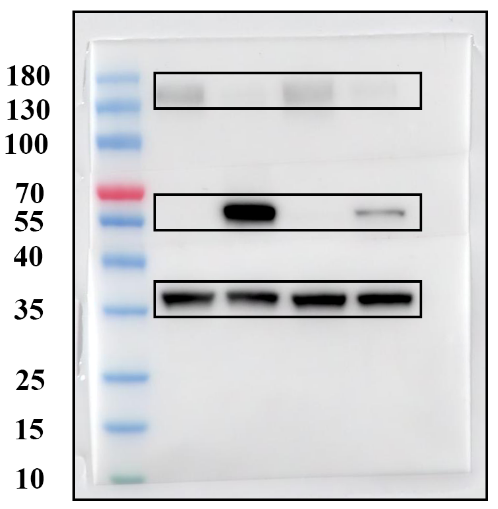


1.
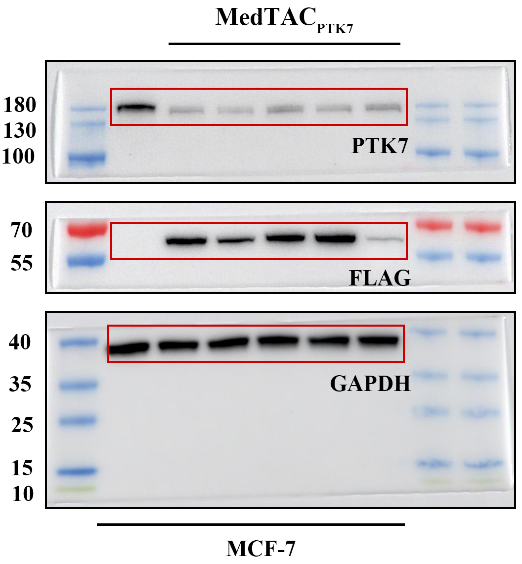
The original western blotting images for Figure 3c.


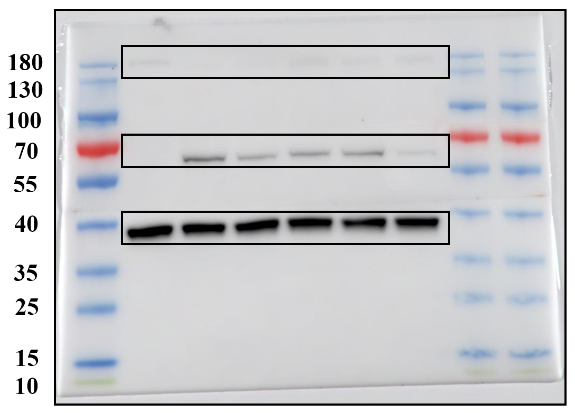


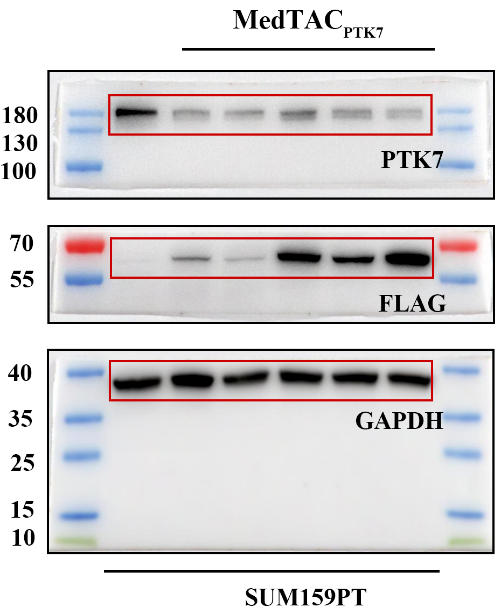

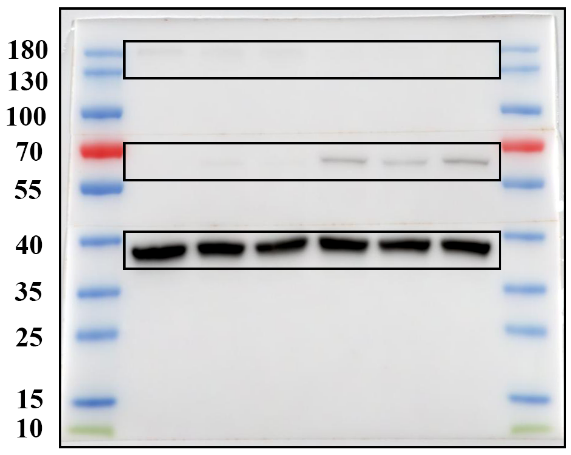


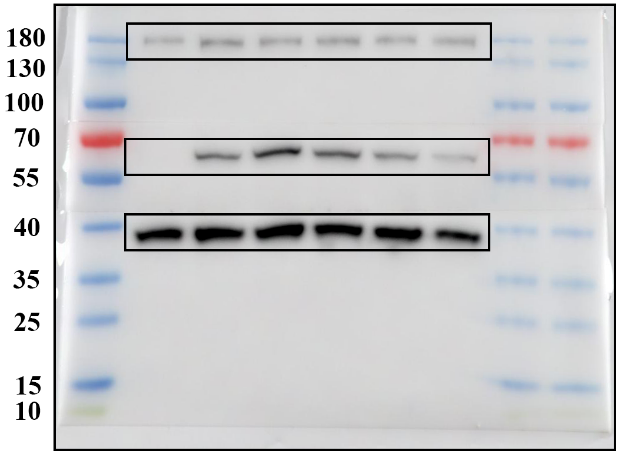

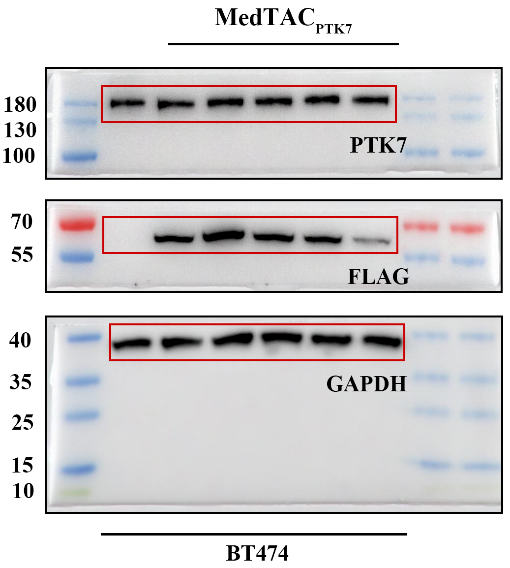


1. The original western blotting images for Figure 4a.


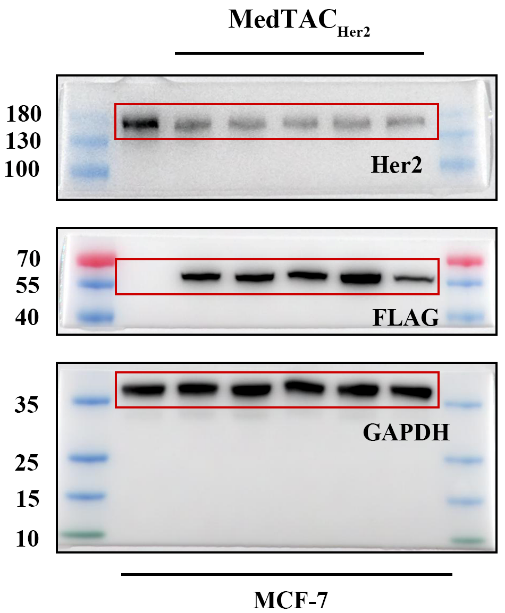

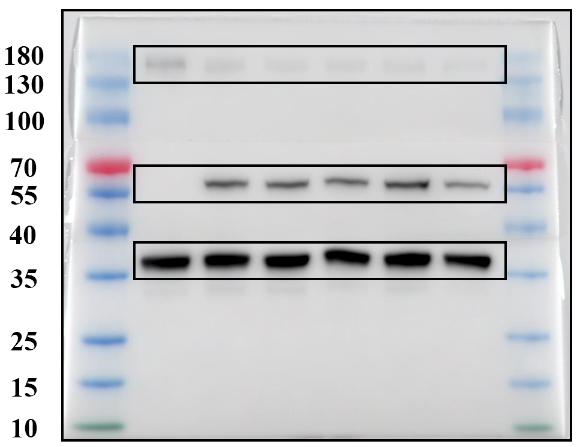


1.
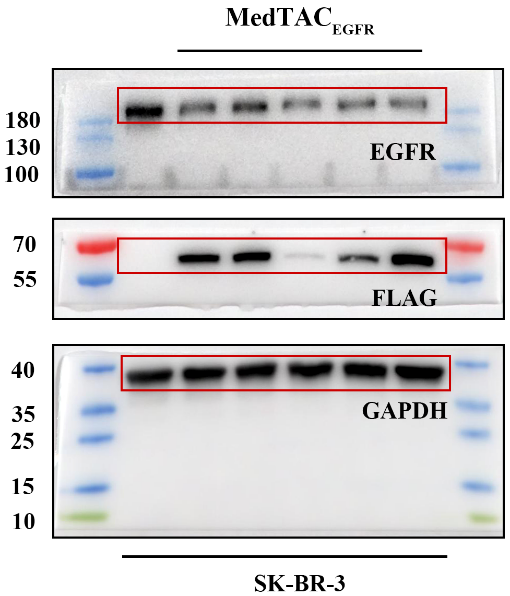
The original western blotting images for Figure 4b.


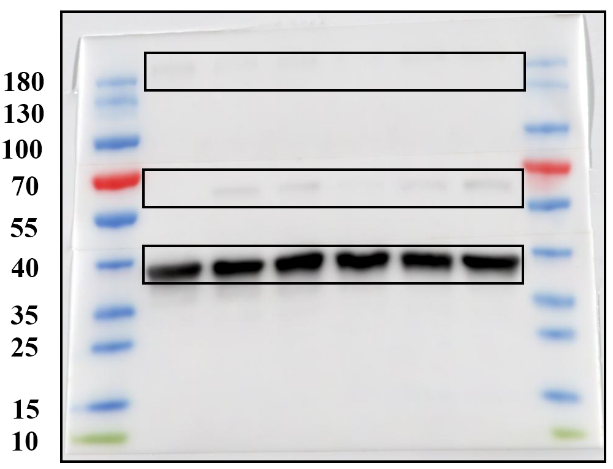


1. The original western blotting images for Figure 4c.


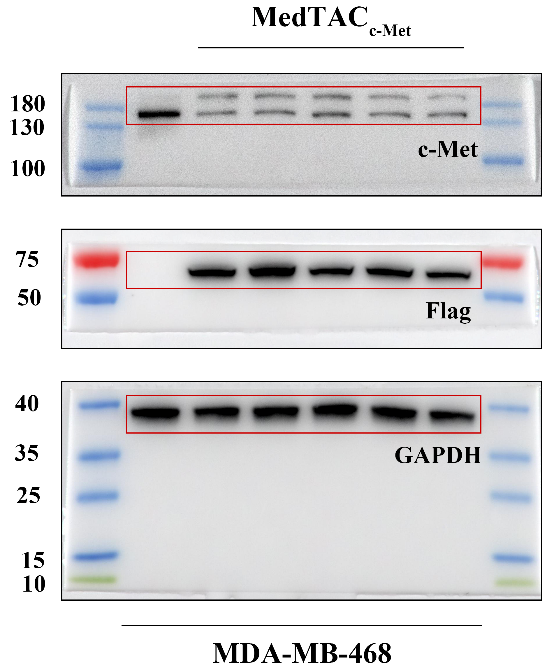

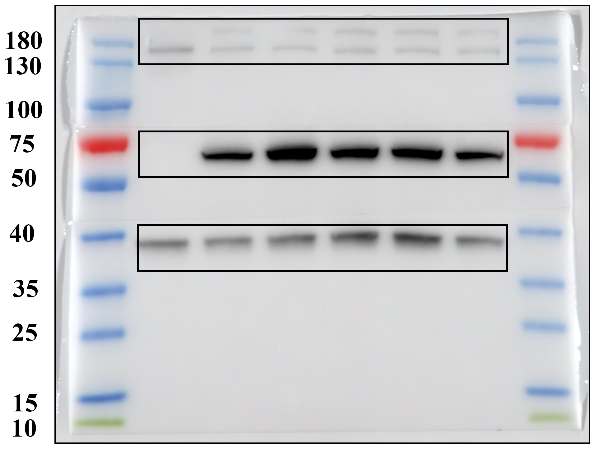


1. The original western blotting images for Figure 6d.


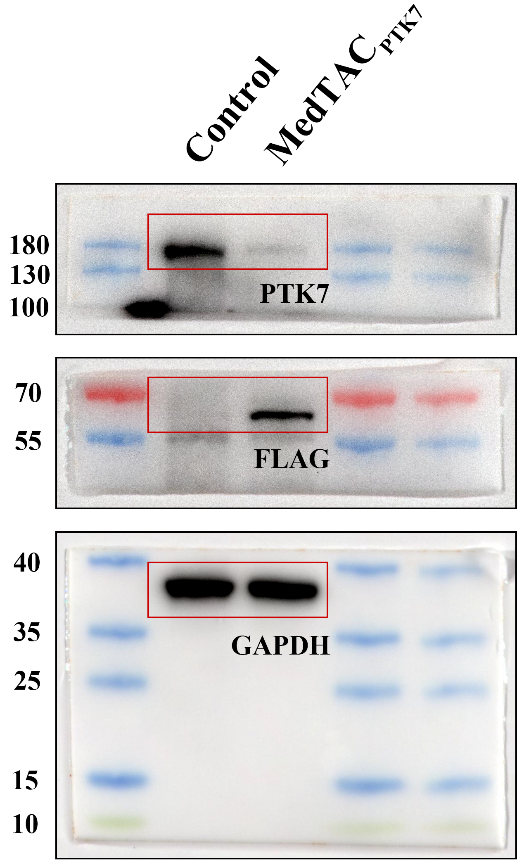


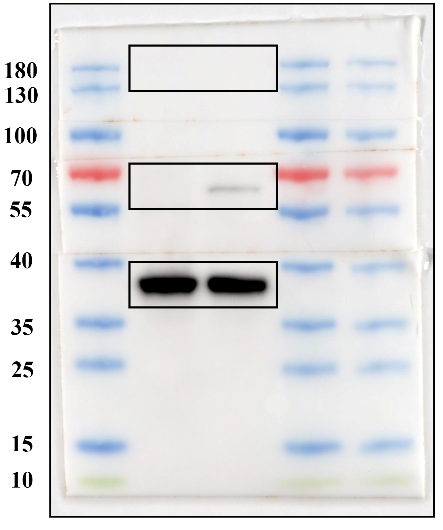


1.
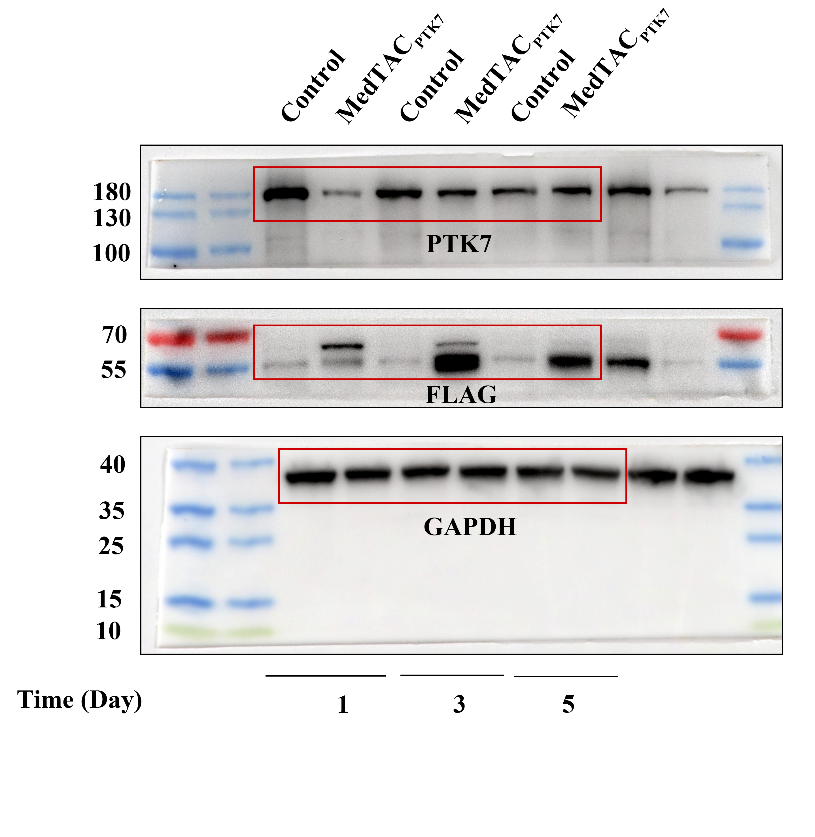
The original western blotting images for Figure 7b.


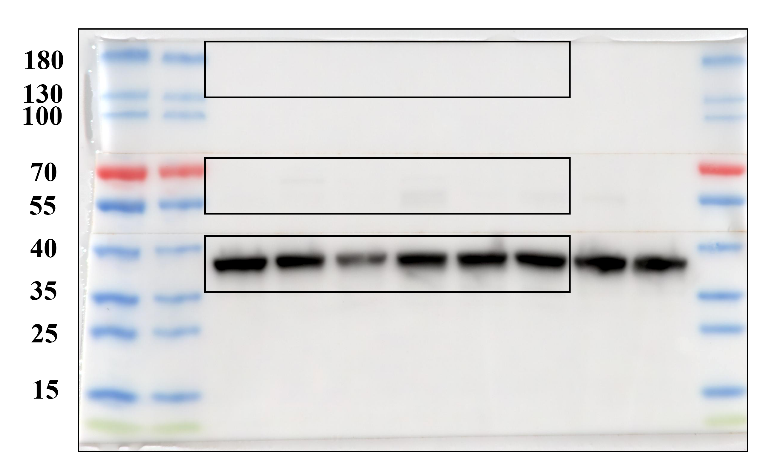


1. The original western blotting images for Figure S4.


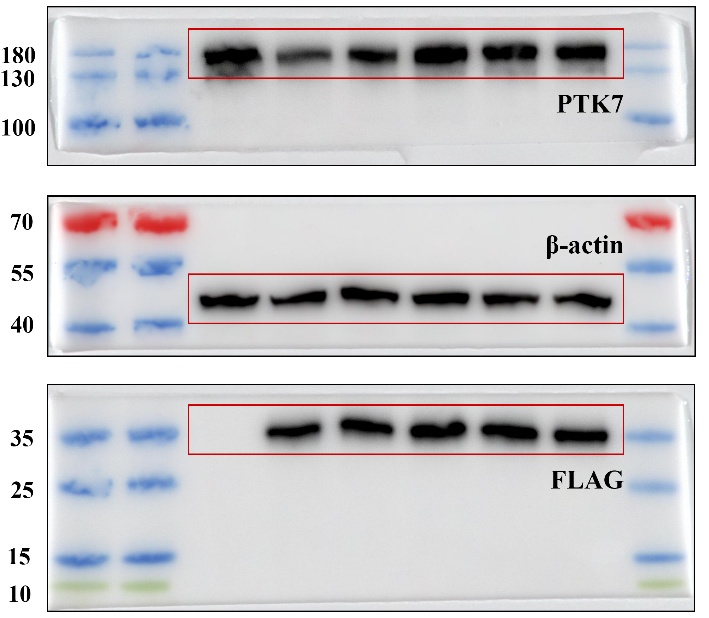


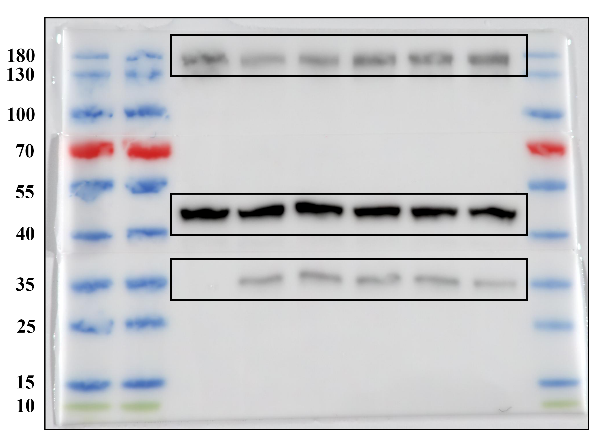


1. The original western blotting images for Figure S7.


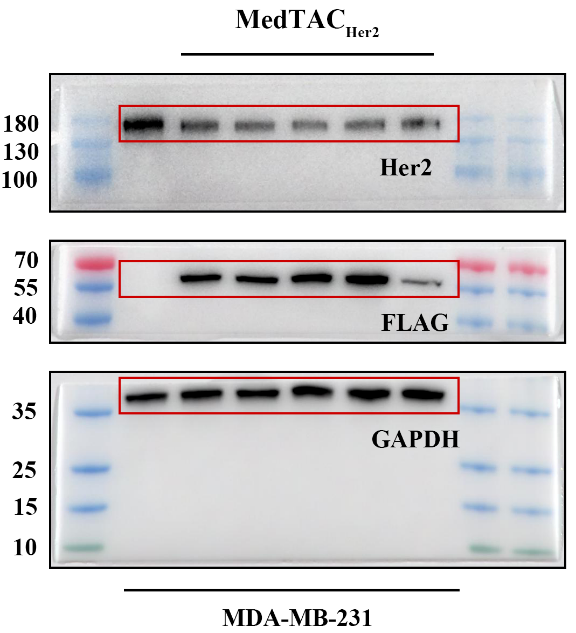


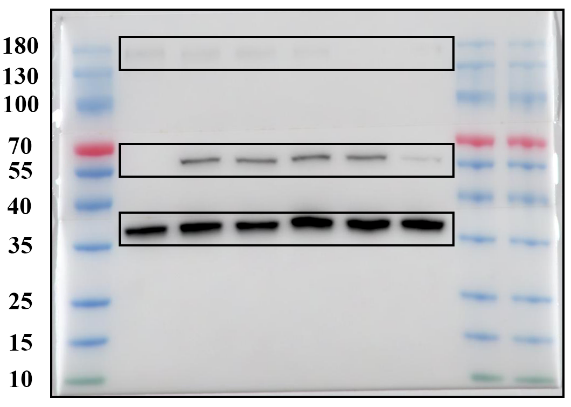


References

[1] S. M. Banik, K. Pedram, S. Wisnovsky, G. Ahn, N. M. Riley, C. R. Bertozzi, *Nature* **2020**, *584*, 291-297.

[2] G. Ahn, S. M. Banik, C. L. Miller, N. M. Riley, J. R. Cochran, C. R. Bertozzi, *Nat Chem Biol* **2021**, *17*, 937-946.

[3] A. D. Cotton, D. P. Nguyen, J. A. Gramespacher, I. B. Seiple, J. A. Wells, *J Am Chem Soc* **2021**, *143*, 593-598.

[4] K. Pance, J. A. Gramespacher, J. R. Byrnes, F. Salangsang, J. A. C. Serrano, A. D. Cotton, V. Steri, J. A. Wells, *Nat Biotechnol* **2023**, *41*, 273-+.

[5] a) D. Zhang, J. Duque-Jimenez, F. Facchinetti, G. Brixi, K. Rhee, W. W. Feng, P. A. Janne, X. Zhou, *Nature* **2024**; b) B. Huang, M. Abedi, G. Ahn, B. Coventry, I. Sappington, C. Tang, R. Wang, T. Schlichthaerle, J. Z. Zhang, Y. Wang, I. Goreshnik, C. W. Chiu, A. Chazin-Gray, S. Chan, S. Gerben, A. Murray, S. Wang, J. O'Neill, L. Yi, R. Yeh, A. Misquith, A. Wolf, L. M. Tomasovic, D. I. Piraner, M. J. Duran Gonzalez, N. R. Bennett, P. Venkatesh, M. Ahlrichs, C. Dobbins, W. Yang, X. Wang, D. D. Sahtoe, D. Vafeados, R. Mout, S. Shivaei, L. Cao, L. Carter, L. Stewart, J. B. Spangler, K. T. Roybal, P. J. Greisen, X. Li, G. J. L. Bernardes, C. R. Bertozzi, D. Baker, *Nature* **2024**.

[6] C. Zhu, W. Wang, Y. Wang, Y. Zhang, J. Li, *Angew Chem Int Ed Engl* **2023**, *62*, e202300694.

[7] J. Zheng, W. He, J. Li, X. Feng, Y. Li, B. Cheng, Y. Zhou, M. Li, K. Liu, X. Shao, J. Zhang, H. Li, L. Chen, L. Fang, *J Am Chem Soc* **2022**, *144*, 21831-21836.
